# Supplementary material for: Assessing COVID-19 pandemic’s impact on essential diabetes care in Manila, the Philippines: A mixed methods study
Source: PLOS Glob Public Health. 2024 Jan 23;4(1):e0002333. doi: 10.1371/journal.pgph.0002333 (PMC10805280; doi:10.1371/journal.pgph.0002333)
Supplement: S1 Appendix — (PDF) [file pgph.0002333.s003.pdf]

S1 Appendix. Likert scales used in the quantitative component.

19a (Av) Pre-COVID, on a scale of 1 to 10 (1 – Very difficult, 10 – Very easy), how easy is it to obtain a consultation slot with this healthcare provider? *[Mula 1 (pinakamahirap) hanggang 10 (pinakamadali), gaano po kadaling makakuha ng appointment para sa inyong check-up para sa diabetes? Paki-rate po mula 1 (pinakamahirap) hanggang 10 (pinakamadali)]*

- ○ 1
- ○ 2
- ○ 3
- ○ 4
- ○ 5
- ○ 6
- ○ 7
- ○ 8
- ○ 9
- ○ 10

19b (Av) At present (in the past 6 months), on a scale of 1 to 10 (1 – Very difficult, 10 – Very easy), how easy is it to obtain a consultation slot with this healthcare provider? *[Mula 1 (pinakamahirap) hanggang 10 (pinakamadali), gaano po kadaling makakuha ng appointment para sa inyong check-up para sa diabetes? Paki-rate po mula 1 (pinakamahirap) hanggang 10 (pinakamadali)]*

- ○ 1
- ○ 2
- ○ 3
- ○ 4
- ○ 5
- ○ 6
- ○ 7
- ○ 8
- ○ 9
- ○ 10

22a (Ac) Pre-COVID, on a scale of 1 to 10 (1 – Very difficult, 10 – Very easy), how easy is it to reach the venue of your DM consultation? *[Mula 1 (pinakamahirap) hanggang 10 (pinakamadali), gaano po kadaling makapunta o makarating sa clinic kung saan kayo nagpapa-check-up para sa diabetes? Paki-rate po mula 1 (pinakamahirap) hanggang 10 (pinakamadali).]*

- ○ 1
- ○ 2
- ○ 3
- ○ 4
- ○ 5
- ○ 6
- ○ 7
- ○ 8
- ○ 9
- ○ 10

22b (Ac) At present (in the past 6 months), on a scale of 1 to 10 (1 – Very difficult, 10 – Very easy), how easy is it to reach the venue of your DM consultation? *[Mula 1 (pinakamahirap) hanggang 10 (pinakamadali), gaano po kadaling makapunta o makarating sa clinic kung saan kayo nagpapa-check-up para sa diabetes? Paki-rate po mula 1 (pinakamahirap) hanggang 10 (pinakamadali).]*

- ○ 1
- ○ 2
- ○ 3
- ○ 4
- ○ 5
- ○ 6
- ○ 7
- ○ 8
- ○ 9
- ○ 10

24a (Af) Pre-COVID, are you able to afford consulting/following-up with your DM healthcare provider as frequently as s/he advised? *(Lagi po bang sapat ang inyong budget para makapagpa-check-up nang kasing dalas ng in-advice ng doktor ninyo?)*

- ○ Never (Hindi)
- ○ Rarely (Bihira)
- ○ Sometimes (Minsan)
- ○ Often (Madalas)
- ○ Always (Lagi)

24b (Af) At present (in the past 6 months), are you able to afford consulting/following-up with your DM healthcare provider as frequently as s/he advised? *(Lagi po bang sapat ang inyong budget para makapagpa-check-up nang kasing dalas ng in-advice ng doktor ninyo?)*

- ○ Never (Hindi)
- ○ Rarely (Bihira)
- ○ Sometimes (Minsan)
- ○ Often (Madalas)
- ○ Always (Lagi)
- ○ Others: ____

33a (Av) Pre-COVID, on a scale of 1 to 10 (1 – Very difficult, 10 – Very easy), how easy was it to find a source for your DM medications? *(Mula 1 (pinakamahirap) hanggang 10 (pinakamadali), gaano po kadaling makahanap ng pagkukuhanan ng gamot para sa diabetes? Paki-rate po mula 1 (pinakamahirap) hanggang 10 (pinakamadali).)*

- ○ 1
- ○ 2
- ○ 3
- ○ 4
- ○ 5
- ○ 6
- ○ 7
- ○ 8
- ○ 9
- ○ 10

33b (Av) At present (in the past 6 months), on a scale of 1 to 10 (1 – Very difficult, 10 – Very easy), how easy was it to find a source for your DM medications? *(Mula 1 (pinakamahirap) hanggang 10 (pinakamadali), gaano po kadaling makahanap ng pagkukuhanan ng gamot para sa diabetes? Paki-rate po mula 1 (pinakamahirap) hanggang 10 (pinakamadali).)*

- ○ 1
- ○ 2
- ○ 3
- ○ 4
- ○ 5
- ○ 6
- ○ 7
- ○ 8
- ○ 9
- ○ 10

36a (Ac) Pre-COVID, on a scale of 1 to 10 (1 – Very difficult, 10 – Very easy), how easy is it to reach the place where you get your DM medications from? *(Mula 1 (pinakamahirap) hanggang 10 (pinakamadali), gaano po kadaling marating o puntahan ang pinagkukuhanan ng gamot ninyo para sa diabetes? Paki-rate po mula 1 (pinakamahirap) hanggang 10 (pinakamadali).)*

- ○ 1
- ○ 2
- ○ 3
- ○ 4
- ○ 5
- ○ 6
- ○ 7
- ○ 8
- ○ 9
- ○ 10

36b (Ac) At present (in the past 6 months), on a scale of 1 to 10 (1 – Very difficult, 10 – Very easy), how easy is it to reach the place where you get your DM medications from? *(Mula 1 (pinakamahirap) hanggang 10 (pinakamadali), gaano po kadaling marating o puntahan ang pinagkukuhanan ng gamot ninyo para sa diabetes? Paki-rate po mula 1 (pinakamahirap) hanggang 10 (pinakamadali).)*

- ○ *1*
- ○ 2
- ○ 3
- ○ 4
- ○ 5
- ○ 6
- ○ 7
- ○ 8
- ○ 9
- ○ 10

38a (Af) Pre-COVID, are you able to afford having enough DM medications so that you could take them regularly, at the prescribed dose? *(Lagi po bang sapat ang inyong budget para regular ninyong mainom ang mga inirisetang gamot sa inyo para sa diabetes?)*

- ◯ Never
- ◯ Rarely
- ◯ Sometimes
- ◯ Often
- ◯ Always

38b (Af) At present (in the past 6 months), are you able to afford having enough DM medications so that you could take them regularly, at the prescribed dose? *(Lagi po bang sapat ang inyong budget para regular ninyong mainom ang mga inirisetang gamot sa inyo para sa diabetes?)*

- ◯ Never
- ◯ Rarely
- ◯ Sometimes
- ◯ Often
- ◯ Always

52a (Av) Pre-COVID, on a scale of 1 to 10 (1 – Very difficult, 10 – Very easy), how easy is it to find a laboratory for your DM tests? *(Mula 1 (pinakamahirap) hanggang 10 (pinakamadali), gaano po kadaling makahanap ng laboratory kung saan pwedeng magpagawa ng mga test para sa diabetes? Paki-rate po mula 1 (pinakamahirap) hanggang 10 (pinakamadali).)*

- ○ 1
- ○ 2
- ○ 3
- ○ 4
- ○ 5
- ○ 6
- ○ 7
- ○ 8
- ○ 9
- ○ 10

52b (Av) At present (in the past 6 months), on a scale of 1 to 10 (1 – Very difficult, 10 – Very easy), how easy is it to find a laboratory for your DM tests? *(Mula 1 (pinakamahirap) hanggang 10 (pinakamadali), gaano po kadaling makahanap ng laboratory kung saan pwedeng magpagawa ng mga test para sa diabetes? Paki-rate po mula 1 (pinakamahirap) hanggang 10 (pinakamadali).)*

- ○ 1
- ○ 2
- ○ 3
- ○ 4
- ○ 5
- ○ 6
- ○ 7
- ○ 8
- ○ 9
- ○ 10

55a (Ac) Pre-COVID, on a scale of 1 to 10 (1 – Very difficult, 10 – Very easy), how easy was it to reach the venue of your DM laboratory test? *(Mula 1 (pinakamahirap) hanggang 10 (pinakamadali), gaano po kadaling mapuntahan o marating ang laboratory kung saan kayo nagpapagawa ng mga test para sa diabetes? Paki-rate po mula 1 (pinakamahirap) hanggang 10 (pinakamadali).)*

- ○ 1
- ○ 2
- ○ 3
- ○ 4
- ○ 5
- ○ 6
- ○ 7
- ○ 8
- ○ 9
- ○ 10

55b (Ac) At present (in the past 6 months), on a scale of 1 to 10 (1 – Very difficult, 10 – Very easy), how easy was it to reach the venue of your DM laboratory test? *(Mula 1 (pinakamahirap) hanggang 10 (pinakamadali), gaano po kadaling mapuntahan o marating ang laboratory kung saan kayo nagpapagawa ng mga test para sa diabetes? Paki-rate po mula 1 (pinakamahirap) hanggang 10 (pinakamadali).)*

- ○ 1
- ○ 2
- ○ 3
- ○ 4
- ○ 5
- ○ 6
- ○ 7
- ○ 8
- ○ 9
- ○ 10

57a (Af) Pre-COVID, are you able to afford having your DM laboratory tests as frequently as your healthcare provider advised? *(Lagi po bang sapat ang inyong budget para maipagawa ninyo ang mga laboratory test para sa diabetes nang kasing dalas ng pag-request nito ng inyong doktor?)*

- ○ Never
- ○ Rarely
- ○ Sometimes
- ○ Often
- ○ Always

57b (Af) At present (in the past 6 months), are you able to afford having your DM laboratory tests as frequently as your healthcare provider advised? *(Lagi po bang sapat ang inyong budget para maipagawa ninyo ang mga laboratory test para sa diabetes nang kasing dalas ng pag-request nito ng inyong doktor?)*

- ○ Never
- ○ Rarely
- ○ Sometimes
- ○ Often
- ○ Always

---

63 (Cv) On a scale of 1 to 10 (1 – Not affected 10 – Very affected), in general, to what extent was your life affected by the COVID lockdown (Enhanced Community Quarantine, ECQ)? *(Mula 1 (hindi naapektuhan) hanggang 10 (lubos na naapektuhan), gaano kalaki ang naging epekto ng COVID sa inyong pamumuhay? Paki-rate po mula 1 (hindi naapektuhan) hanggang 10 (lubos na naapektuhan).)*

- ○ 1
- ○ 2
- ○ 3
- ○ 4
- ○ 5
- ○ 6
- ○ 7
- ○ 8
- ○ 9
- ○ 10
