# Supplementary material for: Assessing COVID-19 pandemic’s impact on essential diabetes care in Manila, the Philippines: A mixed methods study
Source: PLOS Glob Public Health. 2024 Jan 23;4(1):e0002333. doi: 10.1371/journal.pgph.0002333 (PMC10805280; doi:10.1371/journal.pgph.0002333)
Supplement: S2 Appendix — (PDF) [file pgph.0002333.s004.pdf]

S2 Appendix. Interview and focus group discussion guides

A. GUIDE FOR PATIENT INTERVIEWS

Guide for Interviewers
The in-depth interviews are intended to further explore patient's perspectives on DM care and e-health during the COVID pandemic. It will focus on the following areas:
DM care

1. Key changes related to availability, accessibility, and affordability specific to healthcare consultations, medications, and laboratory monitoring
2. Main challenges and coping mechanisms

e-Health to support DM care during COVID

1. Actual experience of using e-health
   - Explore enablers and barriers to the use of e-health
2. Perceived potential uses of e-health moving forward
3. Attitude towards the use of e-health in the "new normal"

Guide Questions:

1. How did the pandemic affect your DM care?
2. What aspects of the pandemic made it more difficult or challenging for you to receive DM care (ex. COVID infection, lack of mobility due to lockdown, lack of finances, lack of previously available services)
3. What challenges did you face in accessing DM care due to the pandemic? How did you cope with these challenges?

Probing Questions:

- During the pandemic, what services related to diabetes care do the primary care centers (local health center/PGH OPD) provide? How do you know about these services?
    - In your opinion, is it necessary to consult/take medications/have laboratory done for your DM during the pandemic?
    - (If previously seeing a doctor) Do you see the need to follow up during the pandemic? Why or why not?
  - If you feel you need urgent/emergent care, where would you access this access this care?
- How did you find DM care providers during the pandemic?
  - Are you willing to get any or all of these services at the local health center/PGH-OPD? Why and why not?
- How did you access DM care during the pandemic?
    - How convenient/easy is it for you to get the care that you need (in terms of distance travelled, waiting time etc.)? Are there other services not available here that you would like to have? What are these services?
    - If you were previously seeing a doctor for your DM, do you have the means to follow up during the pandemic? How?
    - If you feel you need urgent/emergent care, do you have the means to access this care?
  - Do you have to miss work to seek DM care? Why?
- What are the expenses you have relating to care for DM (e.g., drugs, insulin, testing, special equipment or services?
    - How affordable is this care?
    - How has the pandemic affected your ability to pay for DM care?
    - How were you able to afford DM care during this pandemic?
      - Do you have to borrow money, take on additional work, etc.?
- How satisfied are you with the quality of your DM care during the pandemic?
- Do you feel you can trust the services, results, and are happy with how you are treated?
- How did your needs change during the pandemic?

4. Use of eHealth for DM care
    - Explore patients' understanding of eHealth and its role in their DM care prior to, during the pandemic and in the future
    - Explore utilization of eHealth for DM care, as well as challenges/barriers in their use and solutions to overcome these challenges

a. What eHealth/mHealth applications did you use to access DM care BEFORE the pandemic?
    - Why did you use these eHealth applications?
    - What challenges did you encounter when using these applications?

b. What eHealth/mHealth applications did you use to access DM care DURING the pandemic? (Identify which applications they continued to use, and what NEW applications they started to use)
    - Why did you use/continue to use these eHealth applications?
    - What challenges did you encounter when using these applications?
    - Ask also: apps they stopped using during the pandemic and why; which apps were difficult to use

Probing Questions:

- How has eHealth technology improved or hindered your knowledge about the services?
- How has eHealth technology increased or decreased your willingness to get the services here?
- How has eHealth technology made the services more or less convenient for you? What new services, if any, were made available by eHealth?
- How has eHealth technology made the services more affordable?
- How has eHealth technology increased or decreased the quality of care you receive and your level of satisfaction?

c. What do you think the role of eHealth in DM care will be moving forward (e.g. in the next 3 years)?
    a. Which eHealth applications/functions would you like to use/continue using to access DM care beyond the pandemic?
    b. How can eHealth be improved? What adjustments need to be done on existing eHealth applications? What will make it easier for you to use eHealth applications moving forward?

B. GUIDE FOR HEALTHCARE WORKER FOCUS GROUP DISCUSSIONS

Guide for FGD facilitators

Focus group discussions (FGD) with healthcare providers will focus on the individual healthcare provider's experience of rendering DM care during the COVID pandemic. It will focus on the following areas:

DM care

1. Key changes to the management of DM (clinical approach and logistic arrangements) specific to patient interactions and consultations, medication prescribing practices, and diagnostic tests (for work-up/monitoring)
2. Main challenges and coping mechanisms

eHealth to support DM care during COVID

1. Actual experience of using e-health
2. Explore enablers and barriers to the use of e-health
3. Perceived potential uses of e-health moving forward
4. Attitude towards the use of e-health in the "new normal"

Guide Questions:

1. How did the pandemic affect how you deliver DM care?
2. What challenges did you face in providing DM care due to the pandemic? How did you cope with these challenges?
3. How did patient needs change during the pandemic? How were you able to respond to these changes? How successful do you think you were in responding to the needs of your patients?

Probing Questions

Explore available DM services during the pandemic, modifications to existing services or addition of new services as a result of the pandemic and reactions of patients to such changes

- What services do you provide at your facility? How do you inform patients about these services?
- Did these services change during the pandemic? What changes or modifications were implemented as a result of the pandemic?
    - How did you inform/make know to patients that your medical services were available during the pandemic?
    - Was the format of these services (e.g., F2F, online/telemedicine, home service, etc.) during the COVID restrictions acceptable to patients? What were the challenges?
- How convenient/easy is it for patients to get the care that they need (in terms of distance travelled, waiting time etc.)? Are there other services not available here that your patients would like to have? What are these services?
    - Ask about what supplies were available/unavailable to HCPs to enable them to provide care to their patients
- How has the pandemic affected the patients' ability to pay for DM care?
    - How affordable is DM care for patients?
    - Where do they get the funds to pay for DM care?
- How satisfied do you think patients are with the quality of the DM services/care they have received during the pandemic?

eHealth for DM care

Explain eHealth and its examples

eHealth encompasses computer applications, phone applications, access to medical records, access to booking, access to health care provider, communication, booking, self-monitoring applications

4. Did you utilize eHealth to provide DM care? Before and during the pandemic which applications did you use?
5. (If yes) How did eHealth affect provision of DM care during the pandemic?

Probing Questions:

- What are the advantages/disadvantages of using eHealth?

- What are the challenges of using eHealth
- How has eHealth technology improved or hindered patient's knowledge about the services?
- How has eHealth technology made the services more or less convenient for patients? What new services, if any, were made available by eHealth?
- How has eHealth technology affected the cost of services for patients? Has it made more expensive or affordable?
- How has eHealth technology affected the quality of care

6. What do you think the role of eHealth in DM care will be moving forward (e.g., in the next 3 years)? Which eHealth applications/functions would you like to use/continue using to provide DM care beyond the pandemic?

C. GUIDE QUESTIONS FOR HEALTH FACILITY ADMINISTRATOR INTERVIEWS

Guide for Interviewers
Key-informant interviews with healthcare facility administrators will focus on institutional adaptations to deliver DM-related services during the COVID pandemic and utilization of eHealth for service provision:
DM care

1. Key institutional changes to DM care services specific to provision of consults, medications, and laboratory tests
2. Main challenges and how the institution coped

e-Health to support DM care during COVID

1. Explore institutional e-health environment (enablers and barriers) at baseline (not necessarily limited to DM care):
   - Technological environment
   - Organizational environment
   - Human environment
   - Economic environment
2. Use of e-health to facilitate DM care (providing healthcare consults, dispensing medications, facilitating laboratory tests)
3. Perceived potential uses of e-health moving forward
4. Attitude towards the use of e-health in the "new normal"

Guide Questions:
DM care

1. How did the pandemic affect how your health facility delivers DM care?
   - What challenges did you face in ensuring that your health facility is able to provide DM care during the pandemic?
   - How did you cope with these challenges?
2. What institutional changes did you implement to ensure continued provision of medical, diagnostic, and treatment services for DM to patients consulting at your facility?
   - What adjustments to resourcing and staffing (i.e. manpower and work hours) did you have to make?
   - Did you adjust your fees for DM care services? Why or why not?
   - What preparations logistical procedural changes were implemented in use of eHealth applications – such as telemedicine (staffing, infrastructure – computers/internet connectivity/development of facility-based apps)
   - How do you monitor and evaluate effectiveness of these measures?
3. How did patient needs change during the pandemic? How were you able to respond to these changes? How successful do you think you were in responding to the needs of your patients?

eHealth for DM

- <u>BEFORE pandemic</u>
  - What eHealth applications did your facility use, if any, to provide health care in general, and for DM care services in particular?
  - What factors facilitated/enabled the design, implementation, and uptake of eHealth in your facility?
  - What were the barriers to the design, implementation, and uptake of eHealth in your facility?
- <u>DURING pandemic</u>
  - Did your facility use eHealth to provide health services in general, and DM care services in particular, during the pandemic?
  - What eHealth applications did you use/implement?

    - What factors facilitated/enabled the design, implementation, and uptake of eHealth in your facility?
    - What were the barriers to design, implementation, and uptake of eHealth in your facility?
    - What challenges did you face when you implemented eHealth during the pandemic?
    - How did eHeatlh affect the way your health facility provided services for DM care during the pandemic?
- What do you think the role of eHealth in DM care will be moving forward (in the next 3 years)?
    - How about for healthcare in general? What eHealth applications/functions do you think your facility will implement or continue to use beyond the pandemic?
