# Supplementary material for: Assessing COVID-19 pandemic’s impact on essential diabetes care in Manila, the Philippines: A mixed methods study
Source: PLOS Glob Public Health. 2024 Jan 23;4(1):e0002333. doi: 10.1371/journal.pgph.0002333 (PMC10805280; doi:10.1371/journal.pgph.0002333)
Supplement: S2 Data — (PDF) [file pgph.0002333.s006.pdf]

S2 Data. Summary of Codes from Qualitative Thematic Analysis

Category	General Codes	Specific Codes	Frequency of Code	Illustrative quotes
Insights from patients	Impacts of COVID-19 for DM treatment and management	Inaccessible health facilities due to lockdowns/social restrictions	13	When the pandemic started, all those necessary check-ups were totally gone. You know I didn't really know how my diabetes was doing because I wasn't able to go back (to PGH OPD). Even though I know that check-ups and laboratory exams are important, I can't just go out because it was prohibited. It was very difficult during the pandemic.<br><br>I usually had to travel for around 3 hours from Cavite (province outside of Metro Manila) to PGH. Because of the lockdowns, there were no bus or any public transportation available. I didn't have a choice but to just wait it out.
		Lack of available health facilities/healthcare providers	10	I lost touch with my doctor for diabetes because we only get to talk when I visit the hospital. I don't have his contact details.<br><br>The hospital where my doctor works became very strict during the pandemic. We were not allowed to seek consultation. We didn't know where else to go. So all the tests and consultation were suspended for like a year but we made sure we were taking our medication daily.<br><br>There was a time when I was very ill, my family had to send me to a hospital. Turned out my blood sugar was very low. We went to three different public hospitals first, and they all turned us down because they were focused on COVID. We had no choice but to find an alternative that was a private

				facility where I can get accommodated but we had to pay. That wasn't a pleasant experience."
		Affordability concerns from job/employment insecurity	16	My last check-up and laboratory were back in 2019. When the pandemic started, the company I worked for had to lay-off some employees, and I was included. I had a Medicard then (health insurance from work) which I used to finance my health expenses for diabetes. Right now, I am just continuing the Metformin... I'm still not sure when can I have a check-up again because money is an issue. Also, right now you can't just go to the facilities because they require swab tests, that's another thing to be concerned about because you to pay for it.<br><br>To tell you the truth money was really an issue. My two sons who are living with me lost their jobs, but I am very fortunate that my grandchildren are now paying for my laboratory and medicine. However, money is still tight. Sometimes I buy medicine that's insufficient for a week because we had to pay for water and electricity bills.<br><br>It was difficult financially. I had problems securing money for my laboratory exams and medications. My work days weren't as frequent as before and my wife lost her job. Plus, we still had other bills to pay.
	Coping strategies	Intentional medication breaks	7	Aside from my diabetes medicine, I was taking other medicine for my heart and cholesterol. When money is tight, I really had to go on days without medication.<br><br>I used to get free diabetes medicine in community health centers. But with the lockdown, we can't just go out anymore. I had to skip some days.

				Of course, I wanted to take the medicine regularly. It’s important. But I had to take stop taking medication every once in a while.
		Shifting or alternating use of herbal remedies	9	When money is tight and it is difficult to buy medicine, I drink banaba leaves. It’s like a tea. I drink it in mornings and evenings.<br><br>I heard from someone that Metformin can be too much for the body. I was taking the same medicine, the branded one. I wasn’t able to consult with my doctor about the side effects because I had no check-ups. So I decided to stop my Metformin and then I used serpentina and lagundi instead. There are capsule forms but I tried the actual herbs. I boil the leaves and drink them.<br><br>One time I had to self-medicate using malunggay leaves. I was scratching my feet so hard because it was very itchy until I accidentally made a small wound that wouldn’t heal. There were pus coming out. I had to use the malunggay leaves to cover up the wound. It worked. I told about this incident to my doctor when I had an online consultation. He was not pleased about what I did.
		Financial help through social networks	8	My son who lives with me was laid off from work. We had to rely from other family members for my medical expenses.<br><br>My wife and I reached out to our friends to see if can borrow money. Because my diabetes medicine is really important.<br><br>I had to ask around. My friends, family relatives and neighbor if I can borrow money just so I can buy medicine.

	Experience on telemedicine	Advantages of shifting to teleconsultations	7	One thing I liked about telemedicine is that it is convenient. Imagine I didn't have to travel hours and then wait in line for a check-up.<br><br>It was helpful because were able to at least save the money we would use for travel and use it for other things instead like medicine.<br><br>With telemedicine I am able to still have my consultations done at home. At this point my children prefers that for me because I am a senior citizen, I still need to be cautious about COVID-19.
		Disadvantages of shifting to teleconsultations	8	The poor mobile signal can be a problem with telemedicine. One time my teleconsult just ended abruptly.<br><br>I would still prefer for a doctor to actually see me in person so they a better look and sense if there's something wrong with me or my vitals.<br><br>It felt like the teleconsults were being rushed. I didn't feel comfortable to ask questions.
Insights from healthcare providers and administrators	Impact of COVID-19 on DM treatment and management	Temporary suspension of out of patient services	10	We had to restructure our workflow in the hospital to address the COVID-19 pandemic. Many conditions were sort of put aside including DM. For instance, we had to temporarily close our out-patient department to abide by the health protocols.<br><br>Before the pandemic, we had a lot of DM patients going for consultations and laboratory and those patients were all lost

				to follow-up given that we had to suspend our out-patient services.<br><br>We had patients coming in asking for consultation. It was really heartbreaking to tell them that our OPD is temporarily suspended. We would refer them to telemedicine but they would still prefer in-person check-up given that they already traveled to get to the hospital.
		Fear of contracting COVID-19 among patients	5	When they lifted the restrictions, some of our patients were still hesitant to go back even for laboratories because of the fear from getting COVID-19.<br><br>Since the PGH became a COVID referral facility, some of our patients became really reluctant to go back to us even if they wanted to. They would hear in televisions and radios about the surge of COVID-19 cases in PGH.<br><br>We recommend to our patients that they can monitor their blood sugar levels in community health centers. But the fear of getting COVID-19 in health centers also served as a barrier. It was difficult given an advice during teleconsults since we don't have any update about their condition.
	Experience on telemedicine	Advantages of employing telemedicine	12	We were able to restart our OPD operations with telemedicine and reconnect with our previous patients which was a good outcome.<br><br>We were able to meet more patients from other parts of the Philippines and even outside of the country.

				Patients who are compliant with medication and laboratories were able to save resources that they would otherwise spend in travel or food when they go in-person to our out-patient department.
		Disadvantages of employing telemedicine	12	I was hesitant about telemedicine at first because it was difficult to provide instructions especially about insulin. It's important that they really see the demonstration in person.<br><br>With telemedicine, there were observable delays especially with referring patients to other services. Since DM is a multiphasic disease, a patient has to be seen by many specialists. The referrals, the back forth with many doctors was tricky with telemedicine and I think this contributed to delays in patient management.<br><br>One main challenge with telemedicine is the lack of physical examination. Without it, we had to only rely on what patients reported.
