# Supplementary material for: Assessing COVID-19 pandemic’s impact on essential diabetes care in Manila, the Philippines: A mixed methods study
Source: PLOS Glob Public Health. 2024 Jan 23;4(1):e0002333. doi: 10.1371/journal.pgph.0002333 (PMC10805280; doi:10.1371/journal.pgph.0002333)
Supplement: Attachment — Submitted filename: Response to Reviewers.pdf [file pgph.0002333.s007.pdf]

8 August 2023

Prof. Catherine Kyobutungi
Prof. Madhukar Pai
Editors-in-Chief
PLOS Global Public Health

Through: **Nicola L. Hawley**
Academic Editor
PLOS Global Public Health

We thank the editors and reviewers for taking great interest in our work and for providing important and actionable feedback that we hope will help improve our manuscript and make our article meet the current journal standards.

In the following table, we respond to the comments and highlight where we implemented the changes in the revised submission.

Comments	Authors' Responses
**Journal Requirements**	
In the ethics statement in the Methods, you have specified that verbal consent was obtained. Please provide additional details regarding how this consent was documented and witnessed, and state whether this was approved by the IRB.	We provided additional details of the verbal consent process for both quantitative and qualitative components (see **pg 6, ln 6-11 and pg 8, ln 10-11**). The consent procedures are part of the study protocol, approved by the IRB, and we highlighted it in the ethics statement (see **pg 9, ln 24**).
We ask that a manuscript source file is provided at Revision. Please upload your manuscript file as a .doc, .docx, .rtf or .tex.	We have uploaded the manuscript file as .DOCX.
Please provide separate figure files in .tif or .eps format.	We have uploaded Figures 1 and 2 using .TIFF in accordance with journal guidelines.
**Reviewer #1**	
Please define 'covidization' earlier in the paper. Presently it is explained well on page 15, lines 44-45; however, since this phrase is still fairly new to the literature and field, it is recommended this definition be placed earlier in the paper upon the first use of the term.	We included a short description of *covidization* on **pg 14, ln 3-4** upon first use of the term. We purposefully refrained from introducing the concept in earlier sections (e.g., Introduction) because the concept and term emerged as part of the qualitative study, especially from healthcare worker FGDs and administrator interviews. Instead, we provided additional description of the government response to the pandemic in the Introduction (**pg 3, ln 6-19**) to give more context to the phenomenon.
To clarify the explanatory sequential mixed methods design, it would be useful to emphasize that the quantitative data was collected/analyzed first and informed the collection and analysis of the qualitative portion. A figure (Similar to the Figure 1 diagram included would be useful in demonstrating/illustrating the analytic process).	We clarified the description of our analyses, with close attention to how we used the explanatory sequential typology, on **pg 9, ln 1-14**.
As written, the paper leads one to presume that much of the analyses were conducted at very similar time points (this presumption is derived from the statement that participants who completed questionnaires were asked presumably directly afterwards if they wanted to participate in an interview). A strict mixed methods researcher may argue this could fall more into the realm of a convergent parallel design (especially with the emphasis	Thank you for recognizing our conceptualization of the explanatory approach. As suggested, we have reflected the non-classical application of the typology in the limitation on **pg 20, ln 33-39**.

on triangulation of the results). However, there is often flexibility, and as you state, the qualitative portions (including IDIs, KIIs, and FGDs), were used to expand, and explain the quantitative findings as per an explanatory approach. However, the researchers may want to briefly mention this as one minor limitation (that the quantitative results may not have been used sequentially to inform the construction/analysis of the qualitative portion).	
For example, was there a particular rationale for why only n = 1 of the key informant interviews were conducted from the PURE study?	We provided additional information on the considerations in the recruitment for qualitative component on **pg 7, ln 36 to pg 8, ln 11**. We considered the structure of the facility and the staff availability. In this particular instance, there as only one concerned administrator in the health facility serving the PURE study urban community cohort.
Page 4, lines 24-25; it is unclear what the (Barangays 178 to 186) is referring to.	The barangay is to the smallest administrative unit in the Philippines. We rephrased to statement to clarify this on **pg 4, ln 25-26**.
What was the rationale for n = 30 as an ideal sample size?	The ideal sample size of 30 was mentioned based on the proposal of earlier qualitative researchers which we have cited. We clarified this on **pg 7, ln 38**.
What were the screening criteria for inclusion for the IDIs?	Patients were screened for inclusion into IDIs primarily based on their willingness/consent and availability. However, we aimed to provide maximum variation according to age distribution, sex difference, and geographic location. We clarified this on **pg 7, ln 35-37**.
Can greater information/clarity be provided about the data availability of the PURE study? How did the researchers access these data to sample participants?	One of the study participants was also an investigator of PURE study. We sought permission from the PURE study team to access the list of potentially eligible participants in the urban community cohort. The line list of participants was made available to the mixed method study investigators. We clarified this on **pg 5, ln 33-35**.
Page 6: lines 35 – 40; what was the basis for assuming 50% of the individuals would report not having access to diabetes care during the pandemic?	Because we did not have any reliable estimate of population level proportion of individuals not having access to care during the pandemic, we used a conservative estimate of 50%, which would result in larger minimum sample size needed.
Was the assumption of 8% of the cohort having diabetes derived from the included citation?	The assumption of 8% prevalence was based on the national prevalence of high fasting blood glucose levels among Filipino adults residing in urban areas, which was derived from the citation (Expanded National Nutritional Survey 2018). We clarified this on **pg 8, ln 2-5**.
The researchers may want to consider including the interview and/or focus group agendas as supplementary material.	We have included the guides in the submission as **S2 Appendix**.
Page 7, lines 27-28: Since multiple criteria can be used to determine saturation, it may be useful to include what criteria was used here.	We specified the criteria we used to determine saturation on **pg 7, ln 39-40**.
Page 7, lines 40-41; It may be useful to see what the values represented. For example, if 10 is very easy, one can presume that 9 may be 'easy', which leads to questions as to why it would have been defined as problematic.	In the actual questionnaire, we did not provide descriptors between 1 and 10. However, we similarly presumed that two-point difference would be considered problematic. In the original manuscript we described *any value less than 9 to be problematic*. To better clarify the categorization we made, we changed

	it to *any value less than or equal to 8 to be problematic* (**pg 8, ln 19**).
While clearly defined in the manuscript, the number of acronyms used is overwhelming in some sections. It is recommended that alternative phrasing be used.	We have made some adjustments throughout the manuscript to address this (e.g., minimizing use of "DM", "HCWs", and "CHWs" and rephrasing statements to minimize acronyms).
It may help the readability and accessibility of this type of study to a wider audience if the researchers could reorganize the results and discussion sections to be a bit more concise (there is some repetition about covidization especially within these sections).	We have made some adjustments throughout the manuscript to address this. We moved some parts of the discussion to introduction (see **pg 3 ln 10-18**) and omitted repetitive paragraphs (e.g., we removed **pg 18 ln 28-36 of the original manuscript submission**). We also simplified some of the paragraphs in the results and discussion sections.
**Reviewer #2**	
Introduction: more context into how COVID impacted/disrupted health services in the Philippines in particular; there is great data from worldwide effects but want more local information, particularly about diabetes (and other NCDs)	While the evidence on this is sparse, we have restructured the introduction to better highlight these points (see **pg 2, ln 34 to pg 3, ln 19**).
It is unclear what convenience sampling refers to and why participants were recruited in this method. Is the population recruited from PGH OPD equal to those recruited from the PURE cohort? Are these populations the same? What was the follow up on patients ?	The convenience sampling mentioned in the recruitment from PGH OPD primarily refers to the use of line list of patients who were scheduled for follow-up telemedicine consult and to the referral of resident doctors of their patients to the study investigators. 100 of patients were recruited from PURE cohort, and 50 were recruited from PGH OPD. Because of the prevailing restrictions at the time, we put a strong emphasis on the availability of the potential participants and study personnel to collect data, and this was reflected in the recruitment design. The population differed in where they access healthcare, and there was no follow-up for patients recruited in the cross-sectional study. We clarified these issues on **pg 5, ln 40 to pg 6, ln 1-4**.
Exclusion criteria: why was the endocrinology subspecialty clinic excluded? what method was used to recruit hospital admin, nurses, residents?	In this study we focused on primary care diabetes care, so we excluded patients seeking care of specialty clinics. We assumed participants referred to endocrine clinic would have either more difficult to control diabetes, more numerous comorbid illnesses, or more complex conditions that would necessitate more specialized care. We included this explanation on **pg 5, ln 21-23**.
Analysis: Likert scales used should be provided as supplementary information	We have included the Likert scales in the submission as **S1 Appendix**.
Figure 2: what is the y -axis of the graph? what is being defined as a challenge?	The y-axis pertains to the proportion of participants reporting challenges on the specific domain of access and component of care. The challenge pertains to the categorization of the Likert scales into problematic as described in **pg 8, ln 18-19**. We clarified this on **Figure 2** by labelling the y-axis and on the Figure caption on **pg 13**.
Table 5: did patients not report covidization of the healthcare system?	The patients also described their experiences of covidization of healthcare without mentioning the actual term, so we did not initially include it on Table 5. However, upon further discussion, we agree to include on the table on **pg 14**.
Discussion: what constitutes a catastrophic health expenditure? while providing great findings, not getting the author's opinion as to why these changes lead to	We operationally defined catastrophic health expenditure as foregoing necessities and/or borrowing

covidization and greater health burdens; what are the root causes that lead to secondary effects exacerbated by the pandemic?	money to afford any component of diabetes care. We clarified this on **pg 8, ln 23-24**.<br><br>We chose to be conservative in presenting our opinion and instead focused on the study results. However, we emphasized how, based on these results, the government's securitized response to the pandemic may have contributed to the challenges in health on **pg 19, ln 36-45**.

In the following table, we describe the changes and justifications for the changes in the reference list. None of the cited papers have been retracted by their respective journals.

Action	Reference	Justification
Removal	World Health Organization. Third round of the global pulse survey on continuity of essential health services during the COVID-19 pandemic [Internet]. Geneva: World Health Organization; 2022 Feb [cited 2023 Feb 27]. Available from: https://www.who.int/publications-detail-redirect/WHO-2019-nCoV-EHS_continuity-survey-2022.1	Part of the introduction that was removed to improve conciseness
Removal	World Health Organization, International Bank for Reconstruction and Development / The World Bank. Tracking Universal Health Coverage: 2021 Global monitoring report [Internet]. Geneva: World Health Organization; 2021 [cited 2022 Dec 10]. Available from: https://www.who.int/publications-detail-redirect/9789240040618	Part of the introduction that was removed to improve conciseness
Addition	Hapal K. The Philippines' COVID-19 Response: Securitising the Pandemic and Disciplining the Pasaway. Journal of Current Southeast Asian Affairs. 2021 Aug 1;40(2):224–44.	Contextualizes the government response to the pandemic
Addition	Paras AY. An Evolutionary Institutionalist Explanation of the Philippines' Militaristic COVID-19 Approach. Soc. 2022 Aug 1;59(4):359–65.	Contextualizes the government response to the pandemic
Addition	Ulep VG, Paterno A, Uy J, Van VS, Casas L, Tan J. The Impact of the COVID-19 Pandemic on Social Health Insurance Claims for High-Burden Diseases in the Philippines [Internet]. Washington DC: Center for Global Development; 2021 May [cited 2023 Jul 20]. Available from: https://www.cgdev.org/publication/impact-covid-19-pandemic-social-health-insurance-claims-high-burden-diseases-philippines	Study on the impact of the pandemic on healthcare utilization for high-burden diseases in the country
Addition	Uy J, Van VTS, Ulep VG, Bayani DB, Walker D. The Impact of COVID-19 on Hospital Admissions for Twelve High-Burden Diseases and Five Common Procedures in the Philippines: A National Health Insurance Database Study 2019-2020. The Lancet Regional Health – Western Pacific [Internet]. 2022 Jan 1 [cited 2023 Jul 20];18. Available from: https://www.thelancet.com/journals/lanwpc/article/PIIS2666-6065(21)00219-4/fulltext	Study on the impact of the pandemic on healthcare utilization for high-burden diseases in the country
Addition	Dworkin SL. Sample Size Policy for Qualitative Studies Using In-Depth Interviews. Arch Sex Behav. 2012 Dec 1;41(6):1319–20.	Previous literature that suggests the use of 30 as ideal sample size in qualitative studies
Addition	Vasileiou K, Barnett J, Thorpe S, Young T. Characterising and justifying sample size sufficiency in interview-based studies: systematic analysis of qualitative health research over a 15-year period. BMC Medical Research Methodology. 2018 Nov 21;18(1):148.	Previous literature that suggests the use of 30 as ideal sample size in qualitative studies
Addition	Guest G, Namey E, Chen M. A simple method to assess and report thematic saturation in qualitative research. PLOS ONE. 2020 May 5;15(5):e0232076.	Expounds on the use of thematic saturation which we used in the qualitative component

We also report the changes we implemented in the figures and corresponding justifications.

<table>
<tr><th>Fig</th><th colspan="2">Description of Change</th><th>Justification</th></tr>
<tr><td rowspan="2">1</td><td>Original Submission
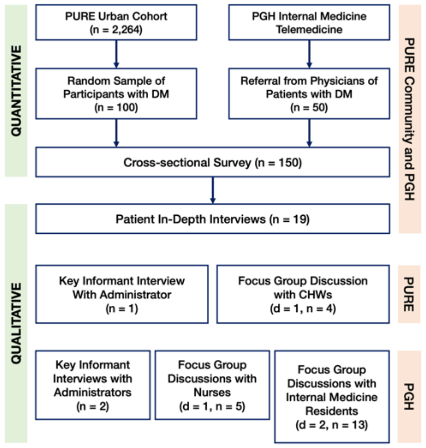
</td><td>Revised Submission
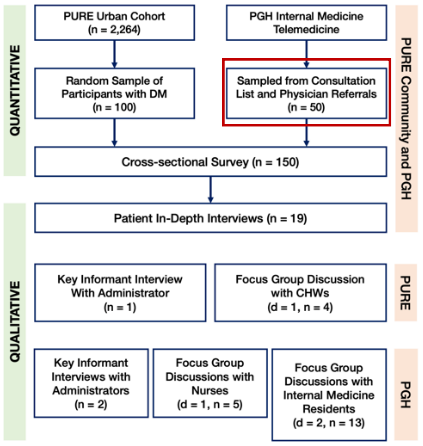
</td><td rowspan="2">The current figure more accurately reflects the recruitment process at PGH OPD (n.b., this was already described in the original submission, see pg 5, ln 36-40 of original manuscript and pg 5, ln 40 to pg 6, ln 4 of the revised manuscript).</td></tr>
<tr><td colspan="2">More complete description of recruitment strategy at PGH OPD</td></tr>
<tr><td rowspan="2">2</td><td>Original Submission
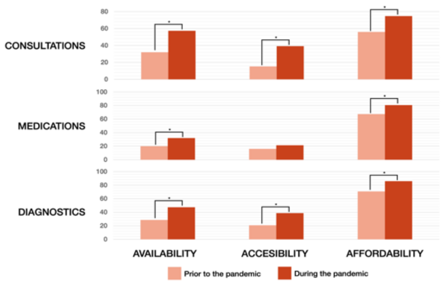
</td><td>Revised Submission
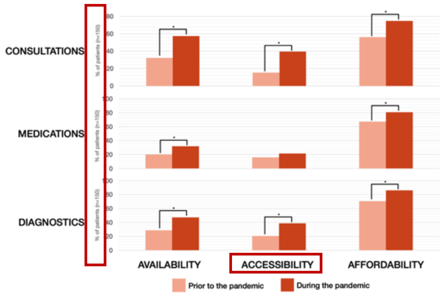
</td><td rowspan="2">The current figure adds clarity on what the numbers on the y-axis mean.</td></tr>
<tr><td colspan="2">Addition of y-axis labels and correction of spelling error</td></tr>
</table>

Finally, we updated the pagination of the STROBE and SRQR checklists to accurately reflect the latest manuscript.

We hope that these responses adequately address the comments and add clarity and quality to our submission. Please address all correspondence concerning this manuscript to **lijing.yan@duke.edu**.

Thank you once again for your kind consideration.

Sincerely,

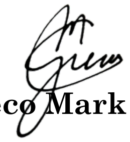

Greco Mark B. Malijan, MD, MSc

On behalf of Prof. Lijing L. Yan (corresponding author)
