# Supplementary material for: Assessing COVID-19 pandemic’s impact on essential diabetes care in Manila, the Philippines: A mixed methods study
Source: PLOS Glob Public Health. 2024 Jan 23;4(1):e0002333. doi: 10.1371/journal.pgph.0002333 (PMC10805280; doi:10.1371/journal.pgph.0002333)
Supplement: Attachment — Submitted filename: PGPH-D-23-00943_R1.pdf [file pgph.0002333.s008.pdf]

PLOS Global Public Health

Assessing COVID-19 Pandemic's Impact on Essential Diabetes Care in Manila, the Philippines: a Mixed Methods Study

--Manuscript Draft--

**Manuscript Number:**	PGPH-D-23-00943R1
**Article Type:**	Research Article
**Full Title:**	Assessing COVID-19 Pandemic's Impact on Essential Diabetes Care in Manila, the Philippines: a Mixed Methods Study
**Short Title:**	Impact of COVID-19 on Essential Diabetes Care in Manila
**Corresponding Author:**	Lijing L Yan<br>Duke University<br>Durham, UNITED STATES
**Order of Authors:**	Greco Mark B. Malijan, MD, MSc
	John Jefferson V. Besa, MD
	Jhaki Mendoza
	Elenore Judy B. Uy, MD, MSc
	Lijing L. Yan, PhD
	Truls Østbye, MD, PhD
	Lia Palileo-Villanueva, MD, MSc
**Keywords:**	diabetes care; COVID-19 pandemic; mixed methods; Philippines; primary care; health systems
**Abstract:**	The COVID-19 pandemic directly increased mortality and morbidity globally. In addition, it has had extensive indirect ill effects on healthcare service delivery across health systems worldwide. We aimed to describe how patient access to diabetes care was affected by the pandemic in Manila, the Philippines. We used an explanatory, sequential mixed method approach including a cross-sectional survey (n = 150) and in-depth interviews of patients (n = 19), focus group discussions of healthcare workers (n = 22), and key informant interviews of health facility administrators (n = 3) from October 2021 to January 2022. Larger proportions of patients reported absence of livelihood (67.3%), being in the lowest average monthly household income group (17.3%), and disruptions in diabetes care (54.0%) during the pandemic. They identified the imposition of lockdowns, covidization of the healthcare system, and financial instability as contributors to the reduced availability, accessibility, and affordability of diabetes-related consultations, medications, and diagnostics. At least a quarter of the patients experienced catastrophic health expenditures across all areas of diabetes care during the pandemic. Most healthcare workers and administrators identified telemedicine as a potential but incomplete tool for reaching more patients, especially those deemed lost to follow-up. In the Philippines, the pandemic negatively impacted access to essential diabetes care.
**Additional Information:**	
**Question**	**Response**
**Financial Disclosure**<br><br>Enter a financial disclosure statement that describes the sources of funding for the work included in this submission and the role the funder(s) played. This includes	The study was funded by the Duke Global Health Institute, SingHealth Duke-National University of Singapore Global Health Institute, and Duke Kunshan University under the collaborative scheme "Health Systems Strengthening to Better Prepare for and Respond to Pandemics". The funders did not play a role in the design, conduct, and analyses of this study.

Powered by Editorial Manager® and ProduXion Manager® from Aries Systems Corporation

grants and any commercial funding of the work or authors.<br><br>This statement will be typeset if the manuscript is accepted for publication.<br><br>*Please review the submission guidelines and the instructions link below for detailed requirements and guidance.*	
**Competing Interests**<br><br>On behalf of all authors, disclose any competing interests that could be perceived to bias this work.<br><br>This statement will be typeset if the manuscript is accepted for publication.<br><br>*Please review the instructions link below and PLOS Global Public Health's competing interests policy to determine what information must be disclosed at submission.*	The authors declare no competing interests.
**Data Availability**<br><br>Before publication, Authors are required to make fully available and without restriction all data underlying their findings. Please see our PLOS Data Policy page for detailed information on this policy.<br><br>A **Data Availability Statement**, detailing where the data can be accessed, is required at first submission. Insert your Data Availability Statement in the box below.<br><br>Please see the data reporting section of our submission guidelines for instructions on what you need to include in your Data Availability Statement.	The data supporting the findings of this study are provided as part of the supplementary information.

This statement will be typeset if the manuscript is accepted for publication.<br><br>*PLOS allows rare exemptions to address legal and ethical concerns. If you have legal or ethical restrictions, please use the box below to detail these in full sentences for the Journal team to consider.*	

Powered by Editorial Manager® and ProduXion Manager® from Aries Systems Corporation

Manuscript Click here to access/download;Manuscript;Manuscript.docx

Assessing COVID-19 Pandemic's Impact on Essential Diabetes Care in Manila, the Philippines: a Mixed Methods Study

Greco Mark B. Malijan[1], John Jefferson V. Besa[2,3], Jhaki Mendoza[2], Elenore Judy B. Uy[4], Lijing L. Yan[5,6*], Truls Østbye[5,7], Lia Palileo-Villanueva[2,3]

[1] San Lazaro Hospital – Nagasaki University Collaborative Research Office, Manila, Philippines

[2] College of Medicine, University of the Philippines Manila, Manila, Philippines

[3] Department of Medicine, Philippine General Hospital, University of the Philippines Manila, Manila, Philippines

[4] Asia Pacific Center for Evidence-based Healthcare, Manila, Philippines

[5] Global Health Research Center, Duke Kunshan University, Jiangsu Province, China

[6] School of Public Health, Wuhan University, Wuhan, China

[7] Department of Family Medicine and Community Health, Duke University, Durham, North Carolina, United States of America

*Corresponding author:
Email address: lijing.yan@duke.edu (LLY)

Abstract

The COVID-19 pandemic directly increased mortality and morbidity globally. In addition, it has had extensive indirect ill effects on healthcare service delivery across health systems worldwide. We aimed to describe how patient access to diabetes care was affected by the pandemic in Manila, the Philippines. We used an explanatory, sequential mixed method approach including a cross-sectional survey (n = 150) and in-depth interviews of patients (n = 19), focus group discussions of healthcare workers (n = 22), and key informant interviews of health facility administrators (n = 3) from October 2021 to January 2022. Larger proportions of patients reported absence of livelihood (67.3%), being in the lowest average monthly household income group (17.3%), and disruptions in diabetes care (54.0%) during the pandemic. They identified the imposition of lockdowns, *covidization* of the healthcare system, and financial instability as contributors to the reduced availability, accessibility, and affordability of diabetes-related consultations, medications, and diagnostics. At least a quarter of the patients experienced catastrophic health expenditures across all areas of diabetes care during the pandemic. Most healthcare workers and administrators identified telemedicine as a potential but incomplete tool for reaching more patients, especially those deemed lost to follow-up. In the Philippines, the pandemic negatively impacted access to essential diabetes care.

Introduction

As early as March 2020, the World Health Organization (WHO) cautioned governments of the ill effects of the coronavirus disease (COVID-19) pandemic on overwhelmed health systems, causing dramatic rise in direct mortality from the outbreak and indirect morality from preventable and treatable conditions in most countries [1]. Communicable disease prevention, reproductive health services, core services for vulnerable populations such as infants and older adults, provision of medications for chronic diseases, critical facility-based therapies, and auxiliary services were identified as high-priority areas to avert indirect morbidities from the pandemic [2]. Despite these early warnings, most health systems worldwide struggled to cope.

Non-communicable disease (NCD) care is increasingly recognized as an important indicator of health system resilience [3]. The WHO reported that up to 75% of member states experienced considerable disruptions to NCD services attributed to the pandemic across all regions and income groups by September 2020 [4]. Human resources allocated to NCD programming either shifted their entire work to COVID-19 services or handled additional responsibilities related to pandemic response. Rehabilitation, hypertension and diabetes care, asthma care, palliative care, and urgent dental care were the services deemed most likely to be disrupted. Moreover, studies on the impact of COVID-19 on health services in the WHO Southeast Asia region found significant reductions in cancer-related

outpatient services, cardiovascular admissions, and respiratory illness diagnoses [5]. Alarmingly, individuals with NCDs such as diabetes, cardiovascular diseases, and cancer are at greater risk of morbidity and mortality from COVID-19 compared to individuals without these conditions [6].

The Philippine government's securitized response to the pandemic was characterized by sustained border and mobility restrictions, stringent penalties against erring individuals, and rapid realignment of health financing and services that reflected the view of COVID-19 being an existential threat [7,8]. These had multifaceted impact on the health system. A systematic literature review of policies and a qualitative study involving system managers in early 2021 found that community quarantines universally impacted health service access and delivery, with greater cascading effects on those needing specialized care [9]. Furthermore, analyses of claims data from PhilHealth, the national health insurance system, found that inpatient care for diabetes, cancer, stroke, and chronic kidney disease had dropped by 57% in 2020 compared to the previous year [10] and that the poorest indigent members experienced the greatest reductions in care [11]. The analyses also found that access limitations were most evident during the implementation of the most stringent lockdown policies.

Levesque and collaborators view access as "the opportunity to reach and obtain appropriate health care services in situations of perceived need for care" [12]. Healthcare access is conceptualized as the interface between the characteristics of providers and health systems and the characteristics of individuals and populations. On the one hand, for optimal healthcare access, services must be approachable, acceptable, available, affordable, and appropriate. On the other hand, individuals must have the ability to perceive, to seek, to reach, to pay, and to engage with existing systems. This framework allows operationalization of the various processes involved in accessing patient-centered care. It offers a lens through which to study both the direct and indirect impact of the pandemic.

Because almost 90% of persons living with diabetes have at least one NCD comorbidity, diabetes serves as an important tracer condition in studying NCD burden [13] and has been used to evaluate health systems and primary care [14,15]. A survey of healthcare professionals from 47 countries found that diabetes care was deemed the primary care service most likely to be affected by the pandemic [16]. Comprehensive diabetes care involves regular interactions with various health service providers at different healthcare levels to facilitate the diagnosis, monitoring, therapy, and rehabilitation and to prevent complications [17]. The difficulties in accessing diabetes care during the pandemic may be a product of delayed care seeking, limited self-care practice, transportation difficulties, outpatient clinic lockdowns, decreased inpatient capacity, and staff and medicine shortages [18].

We aimed to describe how access to diabetes care, as a tracer condition for NCDs in general, was affected by the COVID-19 pandemic in Manila, the Philippines. Specifically, we assessed the availability, accessibility, and

affordability of consultations, medications, and diagnostic procedures associated with diabetes primary care through a mixed methods study.

Materials and Methods

Study Design

We used an explanatory, sequential mixed method approach to assess the impact of COVID-19 on diabetes primary care. We conducted a cross-sectional survey followed by in-depth interviews (IDIs) among patients with diabetes. To corroborate the quantitative and qualitative results from the patient perspective, we conducted focus group discussions (FGDs) and key informant interviews (KIIs) among healthcare providers and health facility administrators. We deemed the mixed method approach to be most appropriate in achieving the overall study aim as it allows triangulation of methodology and perspectives that may better capture co-created realities.

Study Setting

We recruited patient participants from the Prospective Urban Rural Epidemiological (PURE) study and the outpatient department (OPD) of the Philippine General Hospital (PGH).

The PURE study is a large longitudinal epidemiological study examining the relationship of societal influences on lifestyle behaviors, cardiovascular risk factors, and incidence of NCDs [19]. It has enrolled more than 250,000 participants from more than 25 countries globally. In the Philippines, 2,264 participants residing in contiguous communities from Barangays 178 to 186 in Tondo, Manila, are enrolled in the PURE study as part of its urban cohort. The participants mostly seek consultations at the nearby local health centers, which are public primary care facilities that serve an identified catchment area. Each health center is typically staffed by a team of doctor(s), dentist(s), nurses, midwives, and community health workers (CHWs) [20]. Health services such as consultations, selected medications and diagnostic tests, and health education and promotion are provided free at point of care. The local health center also serves as the implementing entity for health programs.

PGH is one of the largest public hospitals in the country and serves as a national tertiary referral center. Located in the City of Manila, its OPD caters to more than 600,000 patients annually. The General Internal Medicine Clinic (GIMC) serves as a typical first point of contact for adult patients seeking care at the OPD. The clinic is staffed by a team of internal medicine resident trainees, nurses, and medical students. In response to the COVID-19 pandemic, PGH implemented a hospital-wide electronic medical record system and teleconsultation services at the OPD.

Participants

For the cross-sectional survey and IDIs with patients, we used the following eligibility criteria.

Inclusion Criteria

1. Adult 18 years or older
2. Diagnosed with diabetes prior to January 2019 (at least one year before the first report of COVID-19 in the Philippines) by a health professional defined as:
    - Patient self-report of physician-adjudicated diagnosis and/or taking any medication for diabetes OR
    - Medical records indicating diagnosis of diabetes
3. Participant of the PURE study OR patient consulting at the PGH GIMC through its telemedicine services

Exclusion Criteria

1. Unable to give informed consent (e.g., severe illness, frailty, dementia, psychological or mental incapacity)
2. Seeking care at a specialty clinic for diabetes care such as the PGH endocrine specialty clinic (n.b., patients referred to the specialty clinic are more likely to have more complex medical conditions necessitating specialized care beyond the level of primary care)

Moreover, we recruited hospital administrators, nurses, internal medicine residents, and community health workers for the qualitative component of the study.

Quantitative Data Collection

Recruitment

Recruitment processes for the cross-sectional study differed between the community and hospital settings, taking into consideration resource limitations and mobility restrictions imposed at the time. We sought permission from the PURE study team to access the list of participants with diabetes in the urban community cohort. We used stratified random sampling, with sex as the stratum-defining variable. A list of ID numbers of eligible participants was used as the sampling frame. Each ID number was assigned using the random number generator function of Microsoft Excel [21]. The assigned random numbers were arranged in ascending order, and participants were recruited consecutively until the desired sample size was reached. To recruit from PGH OPD, we sampled by convenience. We secured a list of patients who were scheduled for follow-up

telemedicine consult from October 2021 to January 2022. We called patients consecutively (i.e., earliest scheduled for a particular day was called first) and recruited those eligible until the desired sample size was reached. We also recruited eligible patients referred to the study by their attending physicians.

Verbal informed consent was discussed and secured from eligible participants. For every consenting patient, an informed consent form was accomplished, indicating the participant's name, date of consent, and name and signature of study personnel who obtained consent. Moreover, a screening log was maintained containing participant name and their decision regarding consent to participate. Once having provided consent, study participants were invited for a telephone, online, or in-person interview, depending on their preference. For in-person interviews, the trained research assistants also obtained a written informed consent prior to the administration of the questionnaire. The study team adhered to minimum health and safety protocols based on prevailing guidelines at the time. The informed consent process for participants enrolled at the PGH GIMC further included a review of medical records to secure accurate information on diagnosis, duration of diabetes, latest laboratory results, and list of prescribed medications.

Instruments

We used a researcher-administered survey questionnaire (S1 Appendix) to characterize the patients' experience of diabetes care before and during the pandemic. The questionnaire was administered once, and participants were asked to recall and report their experiences before and during the pandemic. We operationally defined 'pre-pandemic' as the period prior to January 2020 and 'during the pandemic' as the period in the preceding six months of the study period. Adapting the Levesque framework [12], we asked questions related to demographic and diabetes clinical characteristics at the time of enrollment and to the availability, accessibility, and affordability of healthcare consultations, medications, and laboratory monitoring pre-pandemic and during the pandemic. We operationally defined *availability* as the presence of an identified provider for a particular service, *accessibility* as the physical ability of the person living with diabetes to reach a particular service, and *affordability* as the presence of financial means to avail of the service.

Survey data were directly encoded into electronic data collection forms that had undergone pre-testing. We used password-protected devices exclusively for the study. Data were de-identified during data collection. The authors had no access to information that could identify individual participants. Each participant was assigned a unique study ID, and patient identifiers were stored separately from study participant data. We adhered to existing Philippine data privacy laws and research data management guidelines in the conduct of the study.

Sample Size

We assumed that 50% of individuals with diabetes in the population would report to not have access to DM care during the pandemic. We estimated a population size of 162, assuming an 8% prevalence of diabetes among the 2,264 participants in the PURE community cohort, based on the national prevalence of high fasting blood glucose among adults residing in urban areas [22]. We assessed that the study would require a sample size of 124 for estimating the expected proportion with 5% absolute precision and 95% confidence [23].

Qualitative Data Collection

Instruments

The qualitative component consisted of interviews among patients and healthcare administrators as well as FGDs among healthcare workers. Semi-structured interview and FGD topic guides (S2 Appendix) were developed to contain key questions tailored for each participant group. The IDIs were intended to further explore patients' perspective on diabetes care and management during the COVID-19 pandemic while the FGDs sought to capture the experiences of healthcare workers in providing diabetes care. Interviews among healthcare administrators were also conducted, focusing on institutional adaptations in delivering diabetes-related healthcare services during the COVID-19 pandemic. Two of the investigators (JM and EM) conducted the IDIs and FGDs using the local language (Filipino) to build better rapport and to address limitations of translations.

Researcher Characteristics and Reflexivity

JM and EM are Filipino- and English-speaking local researchers with background in social sciences and experience in doing qualitative health research in the Philippines. Their background provided a non-medical point of view for the study which the study team deemed advantageous as JM and EM prioritized the lived experiences of accessing and delivering care for DM from varying points of view of study participants. This contrasts with the clinical and epidemiological background the rest of the study team provided.

Study size and Recruitment

After the quantitative survey, patients were asked about their willingness to participate further in the qualitative component of the study. Those who expressed interest were screened for inclusion into the IDIs using maximum variation of age distribution, sex, and location. The ideal sample size initially identified for the IDIs was 30 based on earlier literature [24,25]. However, data saturation [26], specifically thematic saturation, where no new concepts were emerging relative to our study population [27], was reached after 15 interviews.

KIIs with health administrators and FGDs among internal medicine residents and nurses were completed at the hospital outpatient clinic. Meanwhile, one FGD with CHWs and an interview with an administrator were conducted in one of the PURE study sites in the City of Manila. In PGH, we purposively selected potential participants to achieve representation based on the type of healthcare provider (e.g., resident doctor, nurse, social worker) and sex difference. Similar criteria were used to recruit from the health center, with additional consideration for staff availability.

For all qualitative study participants, verbal informed consent was discussed and audio recorded before the start of any interview or FGD.

Quantitative Data Analysis

We summarized demographic and clinical characteristics into proportions and used median [interquartile range] to summarize non-normally distributed data. We collapsed Likert scales that were used to characterize availability, accessibility, and affordability of consultations, medications, and laboratory monitoring. The Likert scale had a range of 1 as very difficult and 10 as very easy, and we categorized any value less than or equal to 8 to be problematic. To assess differences in socioeconomic status, diabetes care, and challenges in access to diabetes care pre-pandemic and during COVID-19, we used McNemar's test for paired or dependent nominal data. In assessing catastrophic health expenditure, which we defined as foregoing other necessities or borrowing money to afford a component of diabetes care, we considered those who had no consultation, medication, or laboratory testing in the past six months as separate layers. Hence, we used Chi-squared test to assess the differences in the reported catastrophic health expenditure pre-pandemic and during pandemic. All statistical tests were conducted using 95% confidence level. We performed all statistical tests and visualizations in R version 4.1.2 and RStudio version 2022.12.0+353 [28].

Qualitative Data Analysis Processes

Two investigators (JM and EM) conducted the preliminary analysis. Patient interviews and KIIs lasted around 45-60 minutes while the FGDs lasted about 60-90 minutes. All recordings were transcribed verbatim. The first analytical process involved inductively coding and thematically analyzing all the transcripts written in the local language to identify emerging patterns. The emerging themes were interpreted relative to our conceptual framework to ensure alignment with the study objectives. This step sought to balance the *emic* (participants' perspective) and *etic* (researchers' perspective) standpoints ensuring reflexivity. The second and last analytical process sought to consolidate the emerging themes with the preliminary quantitative findings through a series of discussion among all investigators.

Mixed-methods Triangulation

In accordance with the explanatory sequential typology, the results of the quantitative component partly informed the analysis of the qualitative component. Data triangulation took place at the point of completion of the statistical analysis of the patient cross-sectional survey and during the interpretation of the interviews and FGDs. We compared and contrasted key findings from both qualitative and quantitative data to arrive at points of convergences and divergences. Thereafter, a second round of coding and qualitative analysis using a deductive approach was conducted by two of the researchers (JM and EM) to further validate our key findings.

This pragmatic approach allowed for the qualitative data and its analysis to add depth to the findings of the quantitative data analysis [29]. The final analysis and interpretation of the sequentially collected, integrated data were agreed upon by the whole study team through consensus building meetings.

Reporting

We combined and applied key components of the mixed methods research manuscript checklist by Lee and collaborators [30], the Strengthening the Reporting of Observational Studies in Epidemiology guidelines [31] (S1 Checklist), and the Standards for Reporting Qualitative Research guidelines [32] (S2 Checklist) in preparing this manuscript.

Ethics Statement

The study protocol, study procedures including verbal consent process, and all study materials including consent forms, data collection tools, and interview and FGD guides were reviewed and approved by the University of the Philippines Manila – Research Ethics Board (UPMREB Code: 2021-375-01).

Results

Between October 2021 and January 2022, we recruited and surveyed a total of 100 participants from the PURE community and 50 participants from the PGH OPD (Fig 1). Additionally, 19 patient interviews were conducted from this mix. One interview with health center administrator and one FGD of four CHWs were conducted in the PURE community. Finally, two FGDs involving 13 medical residents, one FGD with five OPD nurses, and two interviews with administrators were conducted in PGH.

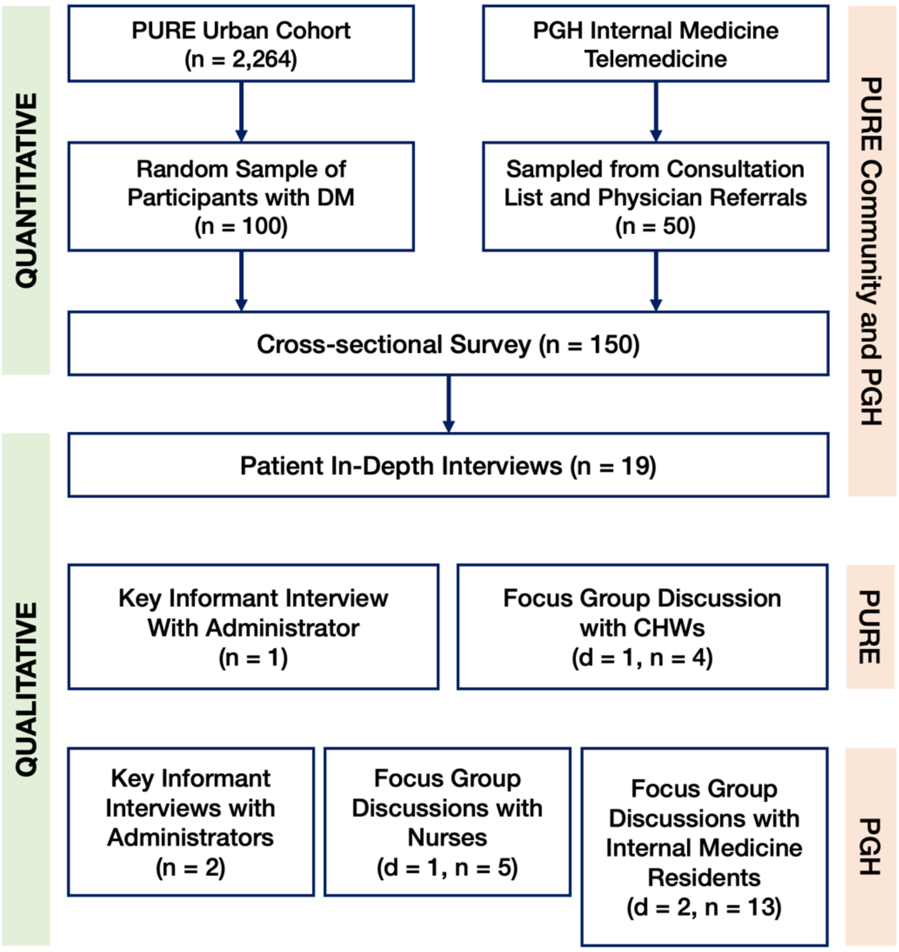

Fig 1. Study participant flow. CHW – community health worker, DM – diabetes mellitus, PGH – Philippine General Hospital, PURE – Prospective Urban and Rural Epidemiological Study; d – number of focus group discussions conducted, n – number of individual study participants included

Cross-sectional Survey Findings

Most of the participants enrolled in the study were middle-aged, female, married, and had completed at least secondary school education (Table 1). The median duration of diabetes diagnosis was 10 years, and the majority had at least one comorbidity, with hypertension being the most frequent.

Table 1. Demographic and clinical characteristics of study participants.

	N = 150 (%)
Age (years)	
Median [interquartile range]	62 [55.0, 68.0]
Age Groups	
19-39 years	3 (2.0)
40-59 years	54 (36.0)
60-69 years	58 (38.7)
70 years and above	35 (23.3)

Sex	
Female	106 (70.7)
Education	
Primary	13 (8.7)
High School	75 (50.0)
Vocational School	11 (7.3)
College/University	51 (34.0)
Marital Status	
Never married	14 (9.3)
Currently married	88 (58.7)
Separated/divorced/annulled	8 (5.3)
Widowed	40 (26.7)
Duration of Diabetes Mellitus (years)	
Median [interquartile range]	10.0 [8.0, 15.0]
Number of Any Comorbid Illness	
Median [interquartile range]	1 [0, 1.0]
Types of Comorbidities	
Hypertension	97 (64.7)
Heart Disease	14 (9.3)
Others	21 (14.0)

Before and during the pandemic, membership in any health insurance scheme and in PhilHealth, the main implementing mechanism for universal health coverage [33], was consistently high (Table 2). However, the proportion of patients with any means of livelihood during the pandemic was significantly lower, and the proportion of patients below the PHP 5,000 monthly household income group during the pandemic were significantly higher than prior to the pandemic. There was no significant difference in household size between periods.

Table 2. Patient socioeconomic status before and during the pandemic.

	Pre-pandemic N (%)	During Pandemic N (%)	p value
Household Size			
≤ 5 members	99 (66.0)	102 (68.0)	0.623
> 5 members	51 (34.0)	48 (32.0)	
Presence of Means of Livelihood			
Yes	85 (56.7)	49 (32.7)	**<0.001**
Average Monthly Household Income (PHP)			
<5,000	9 (6.0)	26 (17.3)	**0.017**
5,000 – 20,000	89 (59.3)	76 (50.7)	
20,001 and above	26 (17.3)	20 (13.3)	
Unknown	26 (17.3)	28 (18.7)	
Membership to any Health Insurance Scheme			
Yes	124 (82.7)	123 (82.0)	0.948
PhilHealth membership			
Yes	115 (76.7)	117 (78.0)	0.617

There were significant differences in the diabetes care received by the patients between time periods (Table 3). Prior to the pandemic, most patients had

been followed in the same healthcare facility for more than two years. However, during the pandemic, more than half of the participants reported having been followed in their healthcare facility for less than a year. Furthermore, 6.7% had to stop taking their medications completely. There was a significant reduction in glycemic control during the pandemic, and almost a quarter of participants did not have any fasting blood sugar (FBS) monitoring.

Table 3. Characteristics of diabetes care before and during the pandemic.

	Pre-pandemic N (%)	During Pandemic N (%)	p value
Duration of Follow-Up in the Healthcare Facility			
< 1 year	7 (4.7)	81 (54.0)	**<0.001**
1 to ≤ 2 years	23 (15.3)	24 (16.0)	
> 2 years	120 (80.0)	42 (28.0)	
Frequency of Medication Intake per Week			
Daily	129 (86.0)	119 (79.3)	**0.008**
Less than daily	21 (14.0)	21 (14.0)	
Stopped medications	0	10 (6.7)	
Controlled Glucose Level during Last Fasting Blood Sugar Testing			
Yes	78 (52.0)	59 (39.3)	**<0.001**
No	59 (39.3)	48 (32.0)	
Unknown	13 (8.7)	9 (6.0)	
No recent testing	0	34 (22.7)	

The proportion of participants who reported challenges in access to consultations, medications, and diagnostic testing during the pandemic increased by up to twice compared to pre-pandemic (Fig 2).

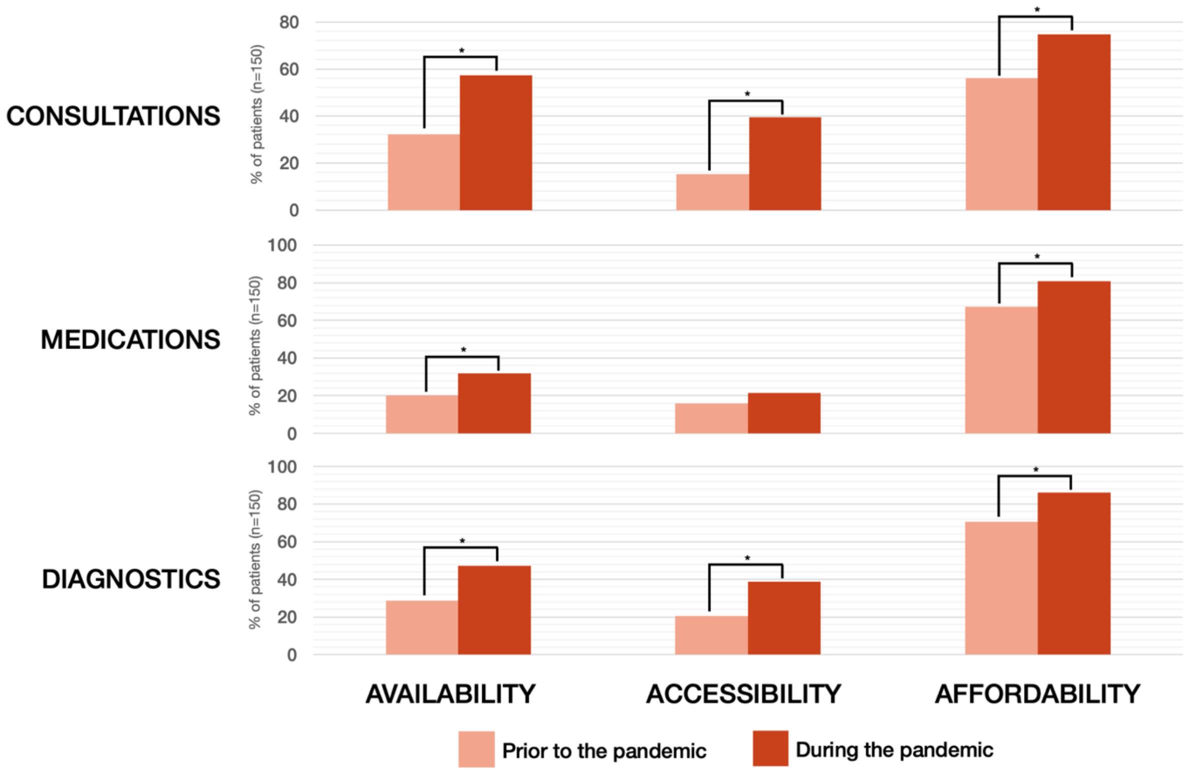

Fig 2. Patient-reported challenges in accessing diabetes care. The proportion of patients reporting challenges, defined as being problematic on categorization of Likert scales, in the respective component of diabetes care and domain of access is represented by the y-axis. Asterisks (*) indicate statistically significant differences ($p < 0.05$) between time periods. There was no correction for multiple comparisons.

Most participants also reported either foregoing necessities or borrowing money to afford consultations, medications, and diagnostic testing, or having no consultation/meds/tests during the pandemic (Table 4).

Table 4. Reported catastrophic health expenditure before and during the pandemic.

	Pre-pandemic N (%)	During Pandemic N (%)	p value
Foregoing Other Necessities or Borrowing Money to Afford Consultation			
Yes	36 (24.0)	40 (26.7)	**<0.001**
No	114 (76.0)	93 (62.0)	
No consult in the last 6 months	0	17 (11.3)	
Foregoing Other Necessities or Borrowing Money to Afford Medications			
Yes	40 (26.7)	50 (33.3)	**<0.001**
No	110 (73.3)	93 (62.0)	
No medication in the last 6 months	0	7 (4.7)	
Foregoing Other Necessities or Borrowing Money to Afford DM Laboratory Tests			
Yes	41 (27.3)	50 (33.3)	**<0.001**
No	109 (72.7)	89 (59.3)	
No testing in the last 6 months	0	11 (7.3)	

Qualitative Findings

The qualitative data revealed three overarching themes that influenced their access and the delivery of services for diabetes care during the pandemic – imposition of mandatory lockdowns, financial instability, and *covidization* (i.e., prioritization of COVID-19 above other societal problems) of the healthcare system (Table 5). These were consistent with our quantitative findings.

Table 5. Summary of qualitative findings.

Themes	Key Findings	Data Source
Imposition of mandatory lockdowns	1. Limited availability of facilities where patients could seek consultations and laboratory examinations affected patients' capacity to monitor their diabetes.	Patients
	2. Reduced social mobility and lack of public transportation options hindered access to health facilities which had impact on patients' capacity to monitor their diabetes and sustain medication.	
Financial instability	1. Losing sources of livelihood during the pandemic tremendously affected patients' capacity to afford consultations, laboratory exams, and medications.	Patients
	2. Trade-offs were present in the form of other competing household expenses which led to suboptimal management for diabetes.	
	3. Limited capacity to afford medication resulted to different coping strategies such as shifting to herbal remedies, taking intentional medication breaks and utilizing social capital to borrow funds for medicine.	
Covidization of the healthcare system	1. The outpatient department, where diabetes was routinely managed, had to temporarily shut down their services for almost a year, effectively restricting access to non-COVID-19 healthcare needs.	Healthcare providers, administrators, and patients
	2. Even with telemedicine, a full assessment for DM patients was difficult to perform without the benefit of physical examination. Additionally, while telemedicine was able to revive provision of outpatient services including diabetes care, healthcare providers felt that many patients were still	

	lost to follow-up due to disparate access to mobile devices.	
	3. Most of the human resources and health supplies were diverted to critical care units to prioritize those with COVID-19. This resulted in delays and suboptimal treatment for admitted patients with complications or severe symptoms from uncontrolled glucose levels.	

Imposition of mandatory lockdowns

The recurrent lockdowns resulted in the suspension of face-to-face consultations and laboratory examinations. Many of the patients in the interview reported having no consultations and examinations done for at least six months, especially during the first two years of the COVID-19 pandemic. Reduced monitoring resulted in uncertainty regarding their diabetes status which they felt compelled to accept. As shared by patient **PG4 (55 years old, female)**:

> *When the pandemic started, all those necessary check-ups were totally gone. You know I didn't really know how my diabetes was doing because I wasn't able to go back (to PGH OPD). Even though I know that check-ups and laboratory exams are important, I can't just go out because it was prohibited. It was very difficult during the pandemic.*

Public transportation became limited as well. Rules on travel were stringent within Metro Manila and even more so for those coming from outside of the region. Despite the eventual ease on heavy lockdowns, travel constraints also had negative impact in accessing facilities for diabetes care.

The restrictions on social mobility coupled with the fear of contracting COVID-19 also impeded some of the patients' access to diabetes care. This was revealed in the account of patient **PG3 (59 years old, female)**:

> *For almost two years, I didn't have any consultations and laboratory exams, but I was taking my medicine continuously. It was the only thing I could do. We were not allowed to go out especially us the elderlies. But I am also afraid to go out even when the most restrictive lockdown was lifted. I still decided not to get any laboratory exams because I don't know, I might get COVID in the diagnostic facility.*

Different experiences regarding access to medications were reported. Some recounted not having any challenges with getting their diabetes medications because pharmacies were still accessible even during lockdowns. While for others, the imposition of granular (i.e., small-scale) lockdowns in certain households precluded any social movement and imposed barriers to medication adherence for certain periods of time.

Financial instability

Many of the patients reported losing their source of livelihood during the pandemic. Such experience was not exclusive to the patients themselves but was also a problem for their family members and relatives who often helped finance diabetes care expenditures. Financial instability greatly affected patients' capacity to afford consultations, laboratory exams, and medications. This is exemplified in the succeeding quote:

> **PU10 (50 years old, male):** *My last check-up and laboratory was back in 2019. When the pandemic started, the company I worked for had to lay-off some employees, and I was included. I had a Medicard then* (health insurance from work) *which I used to finance my health expenses for diabetes. Right now I am just continuing the Metformin… I'm still not sure when can I have a check-up again because money is an issue. Also, right now you can't just go to the facilities because they require swab tests, that's another thing to be concerned about because you to pay for it.*

Meeting the financial demands for diabetes care resulted in trade-offs. Health expenses competed with other household expenses leading to suboptimal self-management for diabetes as shown in the illustrative quotes below:

> **PU16 (73 years old, female):** *To tell you the truth money was really an issue. My two sons who are living with me lost their jobs, but I am very fortunate that my grandchildren are now paying for my laboratory and medicine. However, money is still tight. Sometimes I buy medicine that's insufficient for a week because we had to pay for water and electricity bills.*
>
> **PG7 (45 years old, male):** *It was difficult financially. I had problems securing money for my laboratory exams and medications. My work days weren't as frequent as before and my wife lost her job. Plus, we still had other bills to pay.*

Difficulties in purchasing medications were met with different coping strategies intended to manage diabetes within the best of patients' capabilities. These actions include shifting to herbal remedies and taking intentional medication breaks. Some also utilized their social capital by borrowing money from friends and relatives. The FGD with CHWs from the PURE community also revealed they had to adapt to meet the needs of DM patients within their catchment areas, who mainly relied on free diabetes medication from the local health centers (e.g., house-to-house visit to personally deliver medication to DM patients).

Covidization of healthcare system

The *covidization* of healthcare has been previously described to illustrate how pandemic-related policies dominated and restructured health service delivery and health research [34–36]. It alludes to the outpouring of resources and attention solely to COVID-19 thereby deprioritizing other serious and pressing health concerns such as the management for chronic conditions.

As revealed in the FGDs with HCWs and administrators, the unavailability of non-COVID healthcare services was a major dilemma for continued diabetes

care. Resident doctors from PGH were consistent in reporting that many of their DM patients were unchecked or lost to follow-up during the first year of the pandemic primarily because the OPD had to discontinue services. Many had no contact with their usual DM patients. To some extent the introduction of telemedicine reinstated the delivery of care to OPD patients including those with diabetes. However, healthcare workers still estimated that they were seeing far fewer DM patients than they used to pre-pandemic.

Despite the influx of DM patients who were using teleconsultations, many were still unable to monitor their glucose levels due to social restrictions and lack of funds. Resident doctors agreed that providing medication instructions was one of the major challenges in using telemedicine. Without the benefit of the physical examination, it was more difficult to fully assess the diabetes condition of many patients, especially those who had gone without laboratory testing or had no access to capillary blood glucose monitoring. Instructing patients on insulin use was another struggle for both healthcare providers and patients. Physicians were seeing much more hesitancy among patients towards starting insulin after receiving initial instructions through teleconsultations.

Telemedicine became instrumental in bridging access to diabetes care, but it came with its own challenges. On a micro level, this included inadequate funding to support the sustained operation of telemedicine within the hospital according to administrators. Inequalities regarding access to mobile devices and disparities on technological literacy were also common challenges identified. Hence, while telemedicine was able to revive provision of outpatient services, healthcare workers and administrators felt that many patients were still lost to follow-up due to COVID-19 restrictions.

The FGDs also revealed that most of the human resources and health supplies were diverted to critical care units to prioritize COVID-19 patients. This resulted in delays and suboptimal treatment for inpatients with complications or severe symptoms from uncontrolled glucose levels.

Many patients were also forced to move from one health facility to another to find one that would cater to their non-COVID related concerns. This scenario is described more vividly by patient **PU14 (female, 56 years old)** when she needed emergency care:

> *"There was a time when I was very ill, my family had to send me to a hospital. Turned out my blood sugar was very low. We went to three different public hospitals first, and they all turned us down because they were focused on COVID. We had no choice but to find an alternative that was a private facility where I can get accommodated but we had to pay. That wasn't a pleasant experience."*

Meanwhile, the community doctor and staff members in one of the PURE study sites mentioned having insufficient supply of medication for diabetes and hypertension and further delays in restocking these medicines because of the periodic lockdowns. Additionally, diabetes care services were limited to dispensing

medication. FBS monitoring service was suspended, and the health center staff also took on new COVID-19-related responsibilities such as contact tracing, rapid testing, vaccination, and facilitating of cremation services.

Triangulating Findings

Overall, we found a strong congruence between the quantitative and qualitative findings. The reported limited availability of diabetes care during the pandemic were largely attributed to the *covidization* of the healthcare system. This was especially felt in Metro Manila, which was the region most severely affected by COVID-19 in the country. The reordering of priorities of health facilities and their human resources to COVID-19 activities may have led some patients to discontinue follow-up with their usual diabetes-care provider and seek care in other facilities. These changes may have contributed to the reported increase in the proportion of patients with <1 year follow-up in their most current healthcare facility.

Similarly, the limitations in the accessibility of consultations and laboratory examinations emerged from the imposition of mandatory lockdowns. In the cross-sectional survey, accessibility to medications did not significantly change during the pandemic. However, as shown by the interviews, some patients who experienced granular lockdowns could not reach drug stores, limiting medication adherence. This was reflected in the reduction in daily medication intake of participants during the pandemic.

Finally, the financial instability experienced by patients and their families meant that it was often difficult to afford appropriate diabetes care. Due to loss of livelihood by patients and/or their caregivers, trade-offs including self-management of diabetes were made, often without professional guidance.

Discussion

We aimed to describe the impact of the COVID-19 pandemic on diabetes care in Metro Manila, primarily from the vantage point of patients, as corroborated by healthcare workers and administrators.

We found adverse changes in the socioeconomic status and diabetes care of patients during the pandemic, most evident in greater proportions of individuals in the lowest income group (i.e., PHP 5,000), which was well below the poverty threshold of PHP 12,030 per month for a family of five [37]. Similarly, that a greater proportion of patients had less than a year of follow up in their current facility during the pandemic suggests that they may have felt compelled to change their facility for consultations. The alarming reduction in the number of patients able to perform FBS monitoring also shows disruptions in a key component of diabetes care [17].

The challenges encountered by patients in accessing diabetes care prior to the pandemic were aggravated during the pandemic. Across all domains and areas of diabetes care, more patients reported challenges during the pandemic, but the accessibility of diabetes medications had not significantly changed, likely due to the continued operations of pharmacies during lockdowns. Despite high enrollment (>75%) into PhilHealth and other health insurance schemes, and service provision being free at point of care in PGH and health centers, most of the patients found challenges in the affordability of consultations, diagnostics, and medications. These difficulties were reflected in our finding that about a quarter of patients experienced catastrophic health expenditures even prior to 2020. The affordability of diagnostics during the pandemic was especially problematic. Our findings echo the well-documented dissociation between PhilHealth enrollment and catastrophic health expenditure [38–40].

Studies on the collateral adverse impact of COVID-19 on healthcare in the Philippines are limited. A qualitative study of public health workers, university staff, and administrators explored the impact the pandemic on local health systems and higher education institutions in the country. Routine health programs were found to be inaccessible due to changing priorities and lack of students to augment the health workforce [41]. The results were consistent with previous research in other less-resourced contexts [42,43]. Despite suggesting patient hesitancy to access health facilities as a challenge, these studies did not include the perspectives of healthcare users.

Our study is the first to highlight the patient perspective in assessing the indirect effects of COVID-19 on other health programs in the country. We broke down health service access and delivery into availability, accessibility, and affordability of consultations, diagnostics, and medications to more readily identify areas that can be prioritized for improvement. The use of mixed methods allowed us to better characterize the patient experience beyond the constraints imposed by our deconstruction and conceptualization of "access". Furthermore, the perspectives of healthcare workers and administrators helped identify challenges in the local health centers and those associated with the use of telemedicine in the hospital.

Our work adds evidence from the Philippines, which implemented one of the most restrictive responses to the COVID-19 pandemic, about the indirect impact of global pandemics on health. The probability of another extreme novel pandemic occurring within a lifetime is high [44], and as governments and health systems reckon with the aftermath of COVID-19, our work echoes the need to integrate NCD care into pandemic preparedness efforts. Our work also shows how the government's securitization of health and its response to the pandemic may have exacerbated already existing health inequities and highlights the importance of prioritizing health financing strategies in the country to reduce catastrophic health expenditure and to make healthcare truly accessible to all.

Our study has several limitations. First, the relatively small sample size used in the cross-sectional study was only powered to estimate the proportion of

the population not receiving DM care and not necessarily the difference in outcomes between two time points. Furthermore, we did not adjust our statistical tests for multiple comparisons. However, even with the limited sample size, the results of the quantitative component consistently suggest generally worse conditions during the COVID-19 pandemic, across different access domains and areas of care.

Second, the catchment population of PGH and the urban location of the community cohort may limit the generalizability of our results to other areas in the Philippines. On the one hand, the longer and stricter lockdowns in Metro Manila would seem to impose greater accessibility issues in the region compared to those outside. On the other hand, the provinces and geographically isolated and disadvantaged areas also experienced lockdowns and resource constraints, and patients may have had even greater accessibility issues compared to those in Metro Manila.

Third, our sampling strategy may have introduced selection bias. By including PGH OPD patients and community-dwelling participants already involved in a different study, the study participants were more likely to already have had better access to healthcare and other positive health-reinforcing behaviors than the average patient with DM. Furthermore, because we excluded patients managed by the diabetes specialty clinic who we assumed to have greater DM care needs, our results are more applicable to assess issues in the primary care setting.

Fourth, inherent in our survey questionnaire design is the potential for recall bias, especially regarding the circumstances prior to the pandemic, and recency bias concerning the events during the pandemic. Social desirability bias may have also discouraged participants to admit borrowing money to afford consultation, medications, and laboratory tests; thus, this may underestimate actual catastrophic health expenditure.

Lastly, we iteratively implemented the explanatory, sequential typology such that the cross-sectional study results were not used to inform the construction of the qualitative study guides. Rather, we used the quantitative results to partly inform the analysis of the qualitative portions of the study. We deemed this appropriate in validating our key findings from the patient perspective as corroborated by insights from healthcare workers and administrations.

Conclusion

The imposition of lockdowns, *covidization* of the healthcare system, and financial instability contributed to the reduced availability, accessibility, and affordability of diabetes care-related consultations, laboratory tests, and medications during the COVID-19 pandemic. These combined effects of the pandemic prompted some patients to self-manage their condition, often without

professional guidance. Telemedicine was viewed by healthcare workers and administrators as a promising yet incomplete bridge to reach both new patients and patients lost to follow-up. Our findings highlight the need to integrate NCD care in pandemic preparedness efforts. Our study supports calls to prioritize health financing reforms in the Philippines to prevent catastrophic health expenditure, especially for primary healthcare conditions necessitating routine outpatient care. The reforms are urgently needed now and in preparation for the next pandemic.

Acknowledgement

We thank Dr. Jhoanna Rose Velasquez for contributing to the conceptualization and ethics approval application for the study. We acknowledge the assistance provided by Chareece Mae Zamora-Pudol, Louie Pudol, and Rachel Cabrega in conducting the cross-sectional study. We also acknowledge the assistance provided by Eunice Mallari in conducting and analyzing the qualitative study.

References

1. World Health Organization. Maintaining essential health services: operational guidance for the COVID-19 context: interim guidance, 25 March 2020 [Internet]. Geneva: World Health Organization; 2020 Mar [cited 2023 Feb 27]. Available from: https://apps.who.int/iris/handle/10665/331561

2. World Health Organization. Maintaining essential health services: operational guidance for the COVID-19 context: interim guidance, 1 June 2020 [Internet]. World Health Organization; 2020 [cited 2023 Feb 27]. Report No.: WHO/2019-nCoV/essential_health_services/2020.2. Available from: https://apps.who.int/iris/handle/10665/332240

3. World Health Organization. Regional Office for South-East Asia. Integration of NCD care in emergency response and preparedness [Internet]. World Health Organization. Regional Office for South-East Asia; 2018 [cited 2023 Jul 20]. Available from: https://apps.who.int/iris/handle/10665/272964

4. World Health Organization. The impact of the COVID-19 pandemic on noncommunicable disease resources and services: results of a rapid assessment [Internet]. Geneva: World Health Organization; 2020 [cited 2022 Dec 10]. Available from: https://www.who.int/publications-detail-redirect/9789240010291

5. Gadsden T, Downey LE, Vilas VDR, Peiris D, Jan S. The impact of COVID-19 on essential health service provision for noncommunicable diseases in the South-East Asia region: A systematic review. The Lancet Regional Health - Southeast Asia [Internet]. 2022 Jun 1 [cited 2022 Dec 10];1. Available from:

https://www.thelancet.com/journals/lansea/article/PIIS2772-3682(22)00010-5/fulltext

6. Nikoloski Z, Alqunaibet AM, Alfawaz RA, Almudarra SS, Herbst CH, El-Saharty S, et al. Covid-19 and non-communicable diseases: evidence from a systematic literature review. BMC Public Health. 2021 Jun 5;21(1):1068.

7. Hapal K. The Philippines' COVID-19 Response: Securitising the Pandemic and Disciplining the Pasaway. Journal of Current Southeast Asian Affairs. 2021 Aug 1;40(2):224–44.

8. Paras AY. An Evolutionary Institutionalist Explanation of the Philippines' Militaristic COVID-19 Approach. Soc. 2022 Aug 1;59(4):359–65.

9. Bayani DB, Tan SG. Health Systems Impact of COVID-19 in the Philippines [Internet]. [cited 2023 Feb 8]. Available from: https://www.cgdev.org/publication/health-systems-impact-covid-19-philippines

10. Ulep VG, Paterno A, Uy J, Van VS, Casas L, Tan J. The Impact of the COVID-19 Pandemic on Social Health Insurance Claims for High-Burden Diseases in the Philippines [Internet]. Washington DC: Center for Global Development; 2021 May [cited 2023 Jul 20]. Available from: https://www.cgdev.org/publication/impact-covid-19-pandemic-social-health-insurance-claims-high-burden-diseases-philippines

11. Uy J, Van VTS, Ulep VG, Bayani DB, Walker D. The Impact of COVID-19 on Hospital Admissions for Twelve High-Burden Diseases and Five Common Procedures in the Philippines: A National Health Insurance Database Study 2019-2020. The Lancet Regional Health – Western Pacific [Internet]. 2022 Jan 1 [cited 2023 Jul 20];18. Available from: https://www.thelancet.com/journals/lanwpc/article/PIIS2666-6065(21)00219-4/fulltext

12. Levesque JF, Harris MF, Russell G. Patient-centred access to health care: conceptualising access at the interface of health systems and populations. International Journal for Equity in Health. 2013 Mar 11;12(1):18.

13. An J, Le QA, Dang T. Association between different types of comorbidity and disease burden in patients with diabetes. J Diabetes. 2019 Jan;11(1):65–74.

14. Nolte E, Bain C, McKee M. Diabetes as a Tracer Condition in International Benchmarking of Health Systems. Diabetes Care. 2006 May 1;29(5):1007–11.

15. Lindström K, Berg L, Rylander B, Hagman A, Olsson L, Bengtsson C. A model for quality assessment in primary health care using the tracer condition technique with insulin treated diabetes as one of the tracers. Scandinavian Journal of Primary Health Care. 1997 Jan 1;15(2):92–6.

16. Chudasama YV, Gillies CL, Zaccardi F, Coles B, Davies MJ, Seidu S, et al. Impact of COVID-19 on routine care for chronic diseases: A global survey of views from healthcare professionals. Diabetes & Metabolic Syndrome: Clinical Research & Reviews. 2020 Sep 1;14(5):965–7.

17. American Diabetes Association. Standards of Medical Care in Diabetes—2022 Abridged for Primary Care Providers. Clinical Diabetes. 2022 Jan 1;40(1):10–38.

18. Mohseni M, Ahmadi S, Azami-Aghdash S, Isfahani HM, Moosavi A, Fardid M, et al. Challenges of routine diabetes care during COVID-19 era: A systematic search and narrative review. Primary Care Diabetes. 2021 Dec 1;15(6):918–22.

19. PHRI.ICT W. PURE – Prospective Urban and Rural Epidemiological Study [Internet]. [cited 2022 Dec 11]. Available from: https://www2.phri.ca/pure/

20. Dayrit M, Lagrada L, Picazo O, Pons M, Villaverde M. The Philippines health system review. Health Systems in Transition [Internet]. 2018 [cited 2022 Dec 11];8(2). Available from: https://apps.who.int/iris/handle/10665/274579

21. Microsoft. Microsoft Excel Spreadsheet Software | Microsoft 365 [Internet]. [cited 2022 Apr 19]. Available from: https://www.microsoft.com/en-ww/microsoft-365/excel

22. Department of Science and Technology - Food and Nutrition Research Institute (DOST-FNRI) M. Philippine Nutrition Facts and Figures - 2018 Expanded National Nutrition Survey [Internet]. Manila: DOST-FNRI; 2020 [cited 2023 Feb 8]. Available from: http://enutrition.fnri.dost.gov.ph/site/uploads/2018_ENNS_Facts_and_Figures.pdf

23. Singh ND and M. Sample Size Calculator for Estimating a Proportion [Internet]. [cited 2023 Feb 8]. Available from: https://www.statulator.com/SampleSize/ss1P.html

24. Dworkin SL. Sample Size Policy for Qualitative Studies Using In-Depth Interviews. Arch Sex Behav. 2012 Dec 1;41(6):1319–20.

25. Vasileiou K, Barnett J, Thorpe S, Young T. Characterising and justifying sample size sufficiency in interview-based studies: systematic analysis of qualitative health research over a 15-year period. BMC Medical Research Methodology. 2018 Nov 21;18(1):148.

26. Faulkner SL, Trotter SP. Data Saturation. In: The International Encyclopedia of Communication Research Methods [Internet]. John Wiley & Sons, Ltd; 2017 [cited 2023 Feb 7]. p. 1–2. Available from: https://onlinelibrary.wiley.com/doi/abs/10.1002/9781118901731.iecrm0060

27. Guest G, Namey E, Chen M. A simple method to assess and report thematic saturation in qualitative research. PLOS ONE. 2020 May 5;15(5):e0232076.

28. RStudio Team. RStudio: Integrated Development for R [Internet]. Boston, MA: RStudio, PBC; 2021 [cited 2022 Apr 5]. (Ghost Orchid). Available from: https://www.rstudio.com/

29. Onwuegbuzie AJ, Leech NL. On Becoming a Pragmatic Researcher: The Importance of Combining Quantitative and Qualitative Research Methodologies. International Journal of Social Research Methodology. 2005 Dec 1;8(5):375–87.

30. Lee SYD, Iott B, Banaszak-Holl J, Shih SF, Raj M, Johnson KE, et al. Application of Mixed Methods in Health Services Management Research: A Systematic Review. Med Care Res Rev. 2022 Jun 1;79(3):331–44.

31. von Elm E, Altman DG, Egger M, Pocock SJ, Gøtzsche PC, Vandenbroucke JP, et al. The Strengthening the Reporting of Observational Studies in Epidemiology (STROBE) statement: guidelines for reporting observational studies. PLoS Med. 2007 Oct 16;4(10):e296.

32. O'Brien BC, Harris IB, Beckman TJ, Reed DA, Cook DA. Standards for reporting qualitative research: a synthesis of recommendations. Acad Med. 2014 Sep;89(9):1245–51.

33. Congress of the Philippines. Republic Act No. 7875 | AN ACT INSTITUTING A NATIONAL HEALTH INSURANCE PROGRAM FOR ALL FILIPINOS AND ESTABLISHING THE PHILIPPINE HEALTH INSURANCE CORPORATION FOR THE PURPOSE [Internet]. 7875. Available from: https://www.philhealth.gov.ph/about_us/ra7875.pdf

34. Lasco G, San Pedro J. The 'covidization' of health care | Inquirer Opinion. Inquirer [Internet]. 2020 Jun 7 [cited 2023 Feb 28]; Available from: https://opinion.inquirer.net/130544/the-covidization-of-health-care

35. Pai M. Covidization of research: what are the risks? Nat Med. 2020 Aug;26(8):1159–1159.

36. Alcalde J. Covidization Of Healthcare Leaves Other Diseases Untreated. 2021 Jan 5 [cited 2023 Feb 28]; Available from: https://worldcrunch.com/st/static_newsletter_page

37. Philippine Statistics Authority. Proportion of Poor Filipinos was Recorded at 18.1 Percent in 2021. 2022 Aug 15 [cited 2023 Feb 28]; Available from: https://psa.gov.ph/sites/default/files/3-%28ons-cleared%29_Rev_Press%20Release%20%28for%20posting%29_2021FY%2C%2011Aug2022_wFooter_wag_ONSF-signed.pdf

38. Lasco G, Yu VG, David CC. The Lived Realities of Health Financing: A Qualitative Exploration of Catastrophic Health Expenditure in the

Philippines. Acta Medica Philippina [Internet]. 2022 Jun 29 [cited 2023 Feb 8];56(11). Available from: https://actamedicaphilippina.upm.edu.ph/index.php/acta/article/view/2389

39. Querri A, Ohkado A, Kawatsu L, Remonte MA, Medina A, Garfin AMC. The challenges of the Philippines' social health insurance programme in the era of Universal Health Coverage. Public Health Action. 2018 Dec 12;8(4):175.

40. Bredenkamp C, Buisman LR. Financial protection from health spending in the Philippines: policies and progress. Health Policy and Planning. 2016 Sep 1;31(7):919–27.

41. Maravilla J, Catiwa J, Guariño R, Yap JF, Pagatpatan C, Orolfo DD, et al. Exploring indirect impacts of COVID-19 on local health systems from the perspectives of health workers and higher education stakeholders in the Philippines using a phenomenological approach. The Lancet Regional Health – Western Pacific [Internet]. 2023 Jan 1 [cited 2023 Feb 8];30. Available from: https://www.thelancet.com/journals/lanwpc/article/PIIS2666-6065(22)00200-0/fulltext#seccesectitle0020

42. Singh DR, Sunuwar DR, Shah SK, Karki K, Sah LK, Adhikari B, et al. Impact of COVID-19 on health services utilization in Province-2 of Nepal: a qualitative study among community members and stakeholders. BMC Health Services Research. 2021 Feb 24;21(1):174.

43. Khan MS, Rego S, Rajal JB, Bond V, Fatima RK, Isani AK, et al. Mitigating the impact of COVID-19 on tuberculosis and HIV services: A cross-sectional survey of 669 health professionals in 64 low and middle-income countries. PLoS One. 2021;16(2):e0244936.

44. Marani M, Katul GG, Pan WK, Parolari AJ. Intensity and frequency of extreme novel epidemics. Proceedings of the National Academy of Sciences. 2021 Aug 31;118(35):e2105482118.

Fig 1. Study participant flow.

Click here to access/download;Figure;Fig 1.tiff

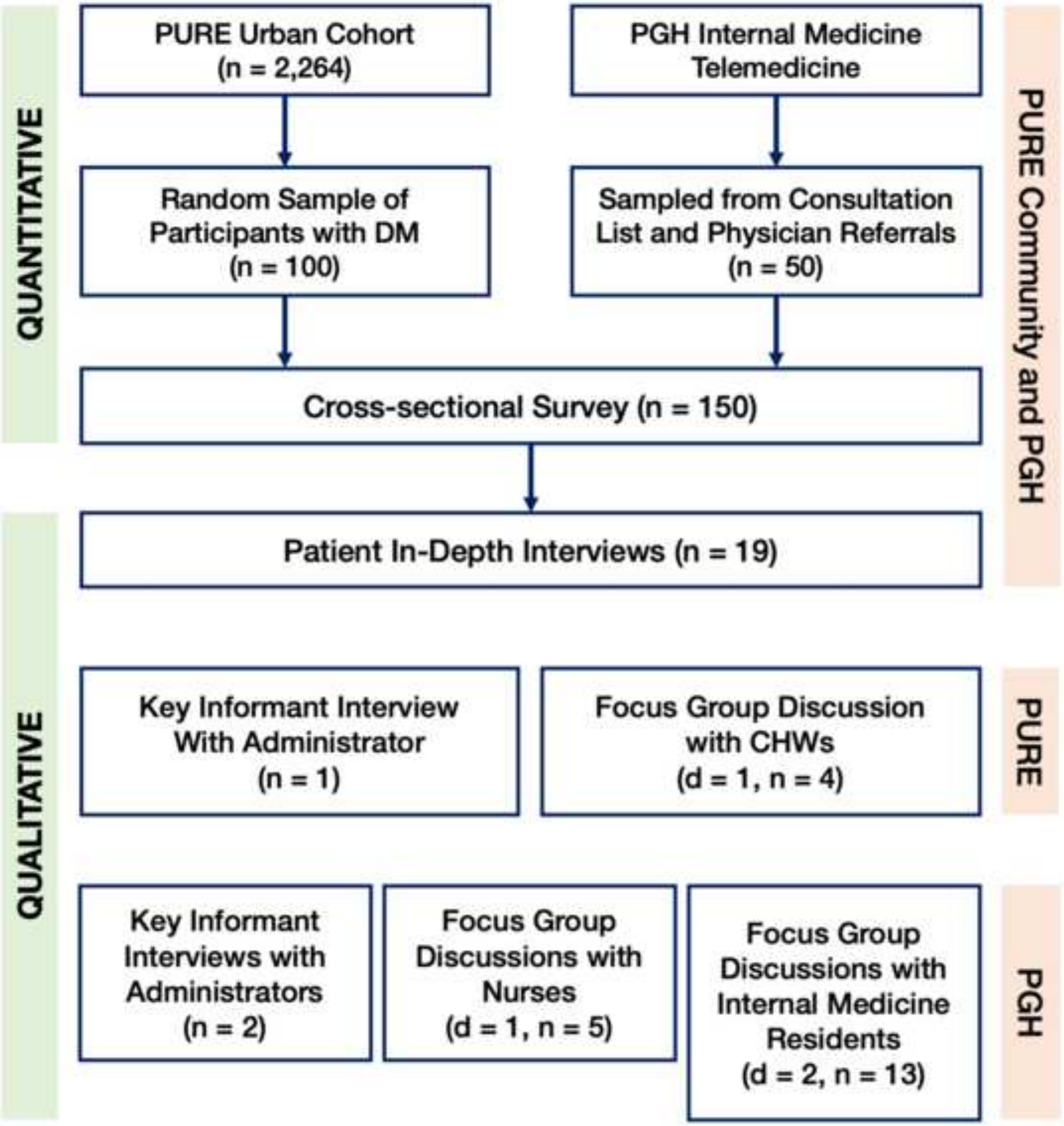

Fig 2. Patient-reported challenges in accessing diabetes care.

Click here to access/download;Figure;Fig 2.tiff

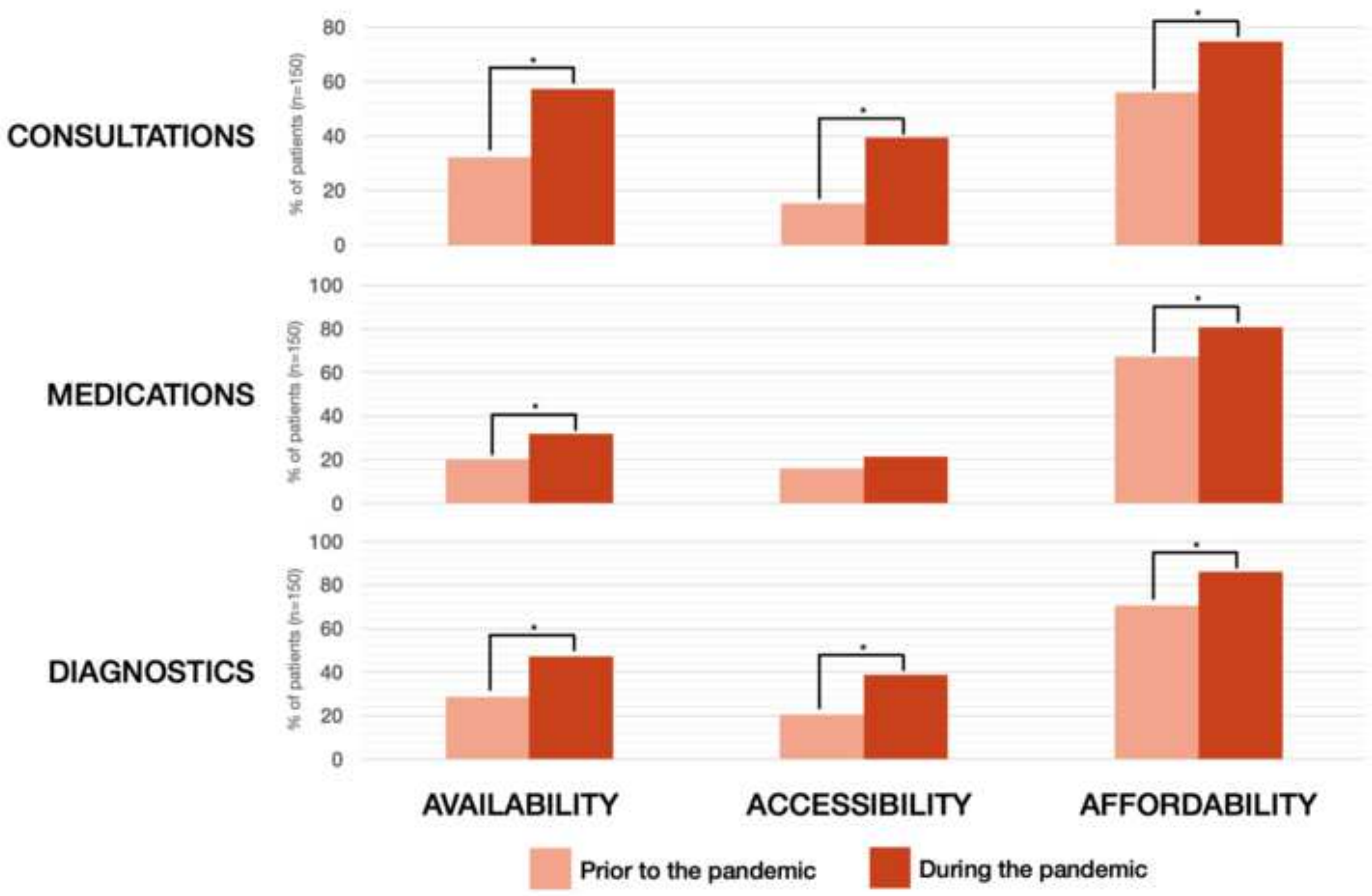

S1 Appendix. Likert scales

Click here to access/download
Supporting Information
S1 Appendix - Likert scales.pdf

S2 Appendix. Interview and FGD guides

Click here to access/download

Supporting Information

S2 Appendix - Interview and FGD guides.pdf

S1 Checklist. Strengthening the reporting of observational studies in epidemiology Checklist

Click here to access/download

Supporting Information

S1 Checklist - STROBE.pdf

S2 Checklist. Standards for reporting qualitative research Checklist

Click here to access/download
Supporting Information
S2 Checklist - SRQR.pdf

Click here to access/download
Supporting Information
S1 Data. Quantitative Component.csv

Click here to access/download
Supporting Information
S2 Data. Qualitative Component.pdf

Assessing COVID-19 Pandemic's Impact on Essential Diabetes Care in Manila, the Philippines: a Mixed Methods Study

Greco Mark B. Malijan[1], John Jefferson V. Besa[2,3], Jhaki Mendoza[2], Elenore Judy B. Uy[4], Lijing L. Yan[5,6*], Truls Østbye[5,7], Lia Palileo-Villanueva[2,3]

[1] San Lazaro Hospital – Nagasaki University Collaborative Research Office, Manila, Philippines

[2] College of Medicine, University of the Philippines Manila, Manila, Philippines

[3] Department of Medicine, Philippine General Hospital, University of the Philippines Manila, Manila, Philippines

[4] Asia Pacific Center for Evidence-based Healthcare, Manila, Philippines

[5] Global Health Research Center, Duke Kunshan University, Jiangsu Province, China

[6] School of Public Health, Wuhan University, Wuhan, China

[7] Department of Family Medicine and Community Health, Duke University, Durham, North Carolina, United States of America

*Corresponding author:
Email address: lijing.yan@duke.edu (LLY)

Abstract

The COVID-19 pandemic ~~has~~ directly increased mortality and morbidity globally. In addition, it has had extensive indirect ill effects on healthcare service delivery across health systems worldwide. We aimed to describe how patient access to diabetes care was affected by the pandemic in Manila, the Philippines. We used an explanatory, sequential mixed method approach including a cross-sectional survey (n = 150) and in-depth interviews of patients (n = 19), focus group discussions of healthcare workers (n = 22), and key informant interviews of health facility administrators (n = 3) from October 2021 to January 2022. ~~Greater~~ Larger proportions of patients reported absence of livelihood (67.3%), ~~belonging to~~being in the lowest average monthly household income group (17.3%), and disruptions in diabetes care (54.0%) during the pandemic. They identified the imposition of lockdowns, *covidization* of the healthcare system, and financial instability as contributors to the reduced availability, accessibility, and affordability of diabetes-related consultations, medications, and diagnostics. At least a quarter of the patients experienced catastrophic health expenditures across all areas of diabetes care during the pandemic. Most healthcare workers and administrators identified telemedicine as a potential but incomplete tool for reaching more patients, especially those deemed lost to follow-up. In the Philippines, the pandemic negatively impacted access to essential diabetes care.

Introduction

~~In~~ As early as March 2020, the World Health Organization (WHO) cautioned governments of the ill effects of the coronavirus disease (COVID-19) pandemic on overwhelmed health systems, causing dramatic rise in direct mortality from the outbreak and indirect morality from preventable and treatable conditions in most countries [1]. ~~Drawing on experience from past outbreak response, the WHO identified communicable~~Communicable disease prevention, reproductive health services, core services for vulnerable populations such as infants and older adults, provision of medications for chronic diseases, critical facility-based therapies, and auxiliary services were identified as high-priority areas to avert indirect morbidities from the pandemic [2]. ~~The latest global pulse survey for essential services found that care for older people remained the most frequently disrupted tracer condition in 2021 and reiterated the need to maintain high-quality access to these services [3].~~Despite these early warnings, most health systems worldwide struggled to cope.

Non-communicable disease (NCD) care is increasingly recognized as an important indicator of health system resilience [3]. The WHO reported that up to 75% of member states experienced considerable disruptions to NCD services attributed to the pandemic across all regions and income groups by September 2020 [4]. Human resources allocated to NCD programming either shifted their entire work to COVID-19 services or handled additional responsibilities related to

pandemic response. Rehabilitation, hypertension and diabetes care, asthma care, palliative care, and urgent dental care were the services deemed most likely to be disrupted. Moreover, studies on the impact of COVID-19 on health services in the WHO Southeast Asia region found significant reductions in cancer-related outpatient services, cardiovascular admissions, and respiratory illness diagnoses [5]. Alarmingly, individuals with NCDs such as diabetes, cardiovascular diseases, and cancer are at greater risk of morbidity and mortality from COVID-19 compared to individuals without these conditions [6].

The Philippine government's securitized response to the pandemic was characterized by sustained border and mobility restrictions, stringent penalties against erring individuals, and rapid realignment of health financing and services that reflected the view of COVID-19 being an existential threat [7,8]. These had multifaceted impact on the health system. A systematic literature review of policies and a qualitative study involving system managers in early 2021 found that community quarantines universally impacted health service access and delivery, with greater cascading effects on those needing specialized care [9]. Furthermore, analyses of claims data from PhilHealth, the national health insurance system, found that inpatient care for diabetes, cancer, stroke, and chronic kidney disease had dropped by 57% in 2020 compared to the previous year [10] and that the poorest indigent members experienced the greatest reductions in care [11]. The analyses also found that access limitations were most evident during the implementation of the most stringent lockdown policies.

Levesque and collaborators view access as "the opportunity to reach and obtain appropriate health care services in situations of perceived need for care" [12]. Healthcare access is conceptualized as the interface between the characteristics of providers and health systems and the characteristics of individuals and populations. On the one hand, for optimal healthcare access to occur, services must be approachable, acceptable, available, affordable, and appropriate. On the other hand, individuals must have the ability to perceive, to seek, to reach, to pay, and to engage with existing systems. This framework provides a comprehensive characterization of patient-centered access and allows operationalization of the various processes involved in accessing patient-centered care. It offers a lens through which to study both the direct and indirect impact of the pandemic.

In response to the rising global burden of non-communicable diseases (NCDs) over the past few decades, countries are increasingly integrating NCD programming into core health services and supporting wider access through universal healthcare coverage [13]. Hence, NCD programming can serve as an important indicator of health system resilience. As early as September 2020, the WHO reported that up to 75% of member states experienced considerable disruptions to NCD services attributed to the pandemic across all WHO regions and income groups [4]. Almost all countries reported that ministry of health staff dedicated to NCD programming either shifted their entire work in support of COVID-19 efforts or engaged in COVID-19 work alongside their NCD-related responsibilities. Rehabilitation, hypertension and diabetes care, asthma care,

~~palliative care, and urgent dental care were the services most likely to be disrupted. A systematic review of studies on the impact of COVID-19 on essential health services in the WHO Southeast Asia region found significant reductions in cancer-related outpatient services, cardiovascular inpatient admissions, and respiratory illness diagnoses and interventions [5]. The review did not include any study on diabetes services. Alarmingly, individuals with NCDs such as cancer, diabetes, cardiovascular diseases, and chronic respiratory illnesses are at a greater risk of morbidity and mortality from COVID-19 compared to individuals without these NCDs [6]. Therefore, ensuring equitable access to NCD services is critical in reducing both the direct and indirect burden of the pandemic.~~

Because almost 90% of persons living with diabetes have at least one NCD comorbidity, diabetes serves as an important tracer condition in studying NCD burden [13] and~~. It~~ has ~~previously~~ been used ~~as a tracer condition~~ to evaluate health systems and primary care [14,15]. ~~Moreover, a~~A survey of healthcare professionals from 47 countries found that diabetes care was deemed the primary care service most likely to be affected by the pandemic ~~compared to other primary care services~~ [16]. Comprehensive diabetes care involves regular interactions with various health service providers at different healthcare levels to facilitate the diagnosis, monitoring, therapy, and rehabilitation and to prevent complications [17]. The difficulties in accessing diabetes care during the pandemic may be a product of delayed care seeking, limited self-care practice, transportation difficulties, outpatient clinic lockdowns, decreased inpatient capacity, and staff and medicine shortages [18].

We aimed to describe how access to diabetes care, as a tracer condition for NCDs in general, was affected by the COVID-19 pandemic in Manila, the Philippines. Specifically, we assessed the availability, accessibility, and affordability of consultations, medications, and diagnostic procedures associated with diabetes primary care through a mixed methods study.

Materials and Methods

Study Design

We used an explanatory, sequential mixed method approach ~~through a cross-sectional survey and in-depth interviews (IDIs)~~ to assess the impact of COVID-19 on diabetes primary care~~ from the patient perspective~~. We conducted a cross-sectional survey followed by in-depth interviews (IDIs) among patients with diabetes. To corroborate the quantitative and qualitative results from the patient perspective, we conducted focus group discussions (FGDs) and key informant interviews (KIIs) among healthcare providers and health facility administrators. We deemed the mixed method approach to be most appropriate in achieving the

overall study aim as it allows triangulation of methodology and perspectives that may better capture co-created realities.

Study Setting

We recruited patient participants from the Prospective Urban Rural Epidemiological (PURE) study and the outpatient department (OPD) of the Philippine General Hospital (PGH).

The PURE study is a large longitudinal epidemiological study ~~study that aims to examine~~examining the relationship of societal influences ~~(built environment, food and nutrition policy, socioeconomic/psychosocial factors and tobacco)~~ on lifestyle behaviors, cardiovascular risk factors, and incidence of NCDs [19]. It has enrolled more than 250,000 participants from more than 25 countries globally. In the Philippines, 2,264 participants residing in contiguous communities from Barangays 178 to 186 in ~~Gagalangin,~~ Tondo, Manila, ~~(Barangays 178 to 186)~~ are ~~, currently~~ enrolled in the PURE study as part of its urban cohort. The participants ~~in the PURE urban cohort~~ mostly seek consultations at the nearby local health centers, which are public primary care facilities that serve an identified catchment area. Each health center is typically staffed by a team of doctor(s), dentist(s), nurses, midwives, and community health workers (CHWs) [20]. Health services such as consultations, selected medications and diagnostic tests, and health education and promotion are provided free at point of care. The local health center also serves as the implementing entity for ~~local and national~~ health programs.

PGH is one of the largest public hospitals in the country and serves as a national tertiary referral center. Located in the City of Manila, its OPD caters to more than 600,000 patients ~~in the OPD~~ annually. The General Internal Medicine Clinic (GIMC) serves as a typical first point of contact for adult patients seeking care at the OPD. The clinic is staffed by a team of ~~of doctors who are~~ internal medicine resident trainees, nurses, and medical students. In response to the COVID-19 pandemic, PGH implemented a hospital-wide electronic medical record system and teleconsultation services at the OPD.

Participants

For the cross-sectional survey and ~~in-depth interviews~~IDIs with patients, we used the following eligibility criteria.

Inclusion Criteria

1. Adult 18 years or older
2. Diagnosed with diabetes prior to January 2019 (at least one year before the first report of COVID-19 in the Philippines) by a health professional defined as:

- Patient self-report of physician-adjudicated diagnosis and/or taking any medication for diabetes OR
- Medical records indicating diagnosis of diabetes

3. Participant of the PURE study OR patient consulting at the PGH GIMC through its telemedicine services

Exclusion Criteria

1. ~~Lack of ability~~Unable to give informed consent (e.g., severe illness, frailty, dementia, psychological or mental incapacity)
2. Seeking care at a ~~diabetes~~ specialty clinic for diabetes care such as the PGH endocrine specialty clinic ~~(e.g., PGH endocrinology subspecialty clinic)~~ (n.b., patients referred to the specialty clinic are more likely to have more complex medical conditions necessitating specialized care beyond the level of primary care)

Moreover, we recruited hospital administrators, nurses, internal medicine residents, and community health workers for the qualitative component of the study.

Quantitative Data Collection

Recruitment

Recruitment processes for the cross-sectional study differed between the community and hospital settings, taking into consideration resource limitations and mobility restrictions imposed at the time. We sought permission from the PURE study team to access the list of participants with diabetes in the urban community cohort. We used stratified random sampling, with sex as the stratum-defining variable~~, to recruit from the PURE urban community cohort~~. ~~We used~~A list of ID numbers~~, arranged in ascending order,~~ of eligible participants was used as the sampling frame. Each ID number was assigned ~~a random number~~ using the random number generator function of Microsoft Excel [21]. The assigned random numbers were arranged in ascending order, and participants were recruited consecutively until the desired sample size was reached. To recruit from PGH OPD, we sampled by convenience. We secured a list of patients who were scheduled for follow-up ~~via telemedicine at the GIMC~~telemedicine consult from October 2021 to January 2022. We called patients consecutively (i.e., earliest scheduled for a particular day was called first) and recruited those eligible until the desired sample size was reached. We also recruited eligible patients referred to the study by their attending physicians.

Verbal informed consent was discussed and secured from eligible participants. For every consenting patient, an informed consent form was accomplished, indicating the participant's name, date of consent, and name and signature of study personnel who obtained consent. Moreover, a screening log was maintained containing participant name and their decision regarding consent to

participate. Once having provided consent, study participants were invited for a telephone, online, or in-person interview, depending on their preference. For in-person interviews, the trained research assistants also obtained a written informed consent prior to the administration of the questionnaire. The study team adhered to minimum health and safety protocols based on prevailing guidelines at the time. The informed consent process for participants enrolled at the PGH GIMC further included a review of medical records to secure accurate information on diagnosis, duration of diabetes, latest laboratory results, and list of prescribed medications.

Instruments

We used a researcher-administered survey questionnaire (S1 Appendix) to characterize the patients' experience of diabetes care before and during the pandemic. The questionnaire was administered once, and participants were asked to recall and report their experiences before and during the pandemic. We operationally defined 'pre-pandemic' as the period prior to January 2020 and 'during the pandemic' as the period in the preceding six months of the study period. Adapting the Levesque framework [12], we asked questions related to demographic and diabetes clinical characteristics at the time of enrollment and to the availability, accessibility, and affordability of healthcare consultations, medications, and laboratory monitoring pre-pandemic and during the pandemic. We operationally defined *availability* as the presence of an identified provider for a particular service, *accessibility* as the physical ability of the person living with diabetes to reach a particular service, and *affordability* as the presence of financial means to avail of the service.

Formatted: Font: Italic

Formatted: Font: Italic

Formatted: Font: Italic

Survey data were directly encoded into electronic data collection forms that had undergone pre-testing. We used password-protected devices exclusively for the study. Data were de-identified during data collection. The authors had no access to information that could identify individual participants. Each participant was assigned a unique study ID, and patient identifiers were stored separately from study participant data. We adhered to existing Philippine data privacy laws and research data management guidelines in the conduct of the study.

Sample Size

We assumed that 50% of individuals with diabetes -in the population would report to not have access to DM care during the pandemic. ~~and~~ We estimated a population size of 162, ~~(~~assuming an 8% prevalence of diabetes ~~[23]~~ among the 2,264 participants in the PURE community cohort, based on the national prevalence of high fasting blood glucose among adults residing in urban areas [22]~~)~~. We assessed that the study would require a sample size of 124 for estimating the expected proportion with 5% absolute precision and 95% confidence [23].

Qualitative Data Collection

Instruments

The qualitative component consisted of interviews among patients and healthcare administrators as well as FGDs among healthcare workers~~ (HCWs). ~~. Semi-structured interview and ~~focus group~~FGD topic guides (S2 Appendix) were developed to contain key questions tailored for each participant group. The IDIs were intended to further explore patients' perspective on diabetes care and management during the COVID-19 pandemic while the FGDs ~~among HCWs~~ sought to capture ~~their ~~the experiences of healthcare workers in providing diabetes care. ~~KIIs ~~Interviews among healthcare administrators were also conducted, focusing on institutional adaptations in delivering diabetes-related healthcare services ~~as a result of~~during the COVID-19 pandemic. Two of the investigators (JM and EM) conducted the IDIs and FGDs using the local language (Filipino) to build better rapport and to address limitations of translations.

Researcher Characteristics and Reflexivity

JM and EM are Filipino- and English-speaking local researchers with ~~a~~ background in social sciences and experience in doing qualitative health research in the Philippines. Their background provided a non-medical point of view for the study which the study team deemed advantageous as JM and EM prioritized the lived experiences of accessing and delivering care for DM from varying points of view of study participants. This contrasts with the clinical and epidemiological background the rest of the study team provided.

Study size and Recruitment

After the quantitative survey, ~~participants ~~patients were asked about their willingness to participate further in the qualitative component of the study. Those who expressed interest were screened for inclusion into the IDIs ~~based on~~using maximum variation of age distribution, sex~~ difference~~, and location. The ideal sample size initially identified for the IDIs was 30 based on earlier literature [24,25]. However, data saturation [26], specifically thematic saturation, where no new concepts were emerging relative to our study population [27], was reached after ~~12 to ~~15 interviews.

KIIs with health administrators and FGDs among internal medicine residents and nurses were completed at ~~PGH GIMC~~the hospital outpatient clinic. Meanwhile, one FGD with CHWs and an~~ KII~~ interview with an administrator were conducted in one of the PURE study sites in the City of Manila. In PGH, we purposively selected potential participants to achieve representation based on the type of healthcare provider (e.g., resident doctor, nurse, social worker) and sex difference. Similar criteria were used to recruit from the health center, with additional consideration for staff availability.

For all qualitative study participants, verbal informed consent was discussed and audio recorded before the start of any interview or FGD.

Quantitative Data Analysis

We summarized demographic and clinical characteristics into proportions and used median [interquartile range] to summarize non-normally distributed data. We collapsed Likert scales that were used to characterize availability, accessibility, and affordability of consultations, medications, and laboratory monitoring. The Likert scale had a range of 1 as very difficult and 10 as very easy, and we categorized any value ~~less than 9~~ less than or equal to 8 to be problematic. To assess differences in socioeconomic status, diabetes care, and challenges in access to diabetes care pre-pandemic and during COVID-19, we used McNemar's test for paired or dependent nominal data. In assessing catastrophic health expenditure, which we defined as foregoing other necessities or borrowing money to afford a component of diabetes care, we considered those who had no consultation, medication, or laboratory testing in the past ~~6~~ six months as separate layers. Hence, we used Chi-squared test to assess the differences in the reported catastrophic health expenditure pre-pandemic and during pandemic. All statistical tests were conducted using 95% confidence level. We performed all statistical tests and visualizations in R version 4.1.2 and RStudio version 2022.12.0+353 [28].

Qualitative Data Analysis Processes

Two investigators (JM and EM) conducted the preliminary analysis. Patient interviews and KIIs lasted around 45-60 minutes while the FGDs lasted about 60-90 minutes. All recordings were transcribed verbatim. The first analytical process involved inductively coding and thematically analyzing all the transcripts written in the local language to identify emerging patterns. The emerging themes were interpreted relative to our conceptual framework to ensure alignment with the study objectives. This step sought to balance the *emic* (participants' perspective) and *etic* (researchers' perspective) standpoints ensuring reflexivity ~~during the initial analytical process~~. The second and last analytical process sought to consolidate the emerging themes with the preliminary quantitative findings through a series of discussion among all ~~the authors~~investigators.

Mixed-methods Triangulation

In accordance with the explanatory sequential typology, the results of the quantitative component partly informed the analysis of the qualitative component. Data triangulation took place at the point of completion of the statistical analysis of the ~~quantitative component~~patient cross-sectional survey and during the ~~analysis and~~ interpretation of the ~~qualitative component~~interviews and FGDs. ~~through consensus building among the investigators. This involved~~We ~~comparing~~ compared and ~~contrasting~~ contrasted key findings from both qualitative and quantitative data to arrive at points of convergences and divergences. Thereafter,

a second round of coding and qualitative analysis using a deductive approach was conducted by two of the ~~authors~~ researchers (JM and EM) to further validate our key findings.

This pragmatic approach allowed for the qualitative data and its analysis to add depth to the findings of the quantitative data analysis [29]. The final analysis and interpretation of the sequentially collected, integrated data were agreed upon by the whole study team through consensus building meetings.

Reporting

We combined and applied key components of the mixed methods research manuscript checklist by Lee and collaborators [30], the Strengthening the Reporting of Observational Studies in Epidemiology guidelines [31] (S1 Checklist), and the Standards for Reporting Qualitative Research guidelines [32] (S2 Checklist) in preparing this manuscript.

Formatted: Indent: First line: 0.5"

Ethics Statement

The study protocol, study procedures including verbal consent process, and all study materials including consent forms, data collection tools, and interview and FGD guides were reviewed and approved by the University of the Philippines Manila – Research Ethics Board (UPMREB Code: 2021-375-01).

Results

Between October 2021 and January 2022, we recruited and surveyed a total of 100 participants from the PURE community and 50 participants from the PGH ~~GIMC~~ OPD (Fig 1). Additionally, 19 patient ~~IDIs~~ interviews were conducted from this mix. One ~~KII~~ interview with health center administrator and one FGD of four CHWs were conducted in the PURE community. Finally, two FGDs involving 13 medical residents, one FGD with five OPD nurses, and two ~~KII~~ interviews with administrators were conducted in PGH.

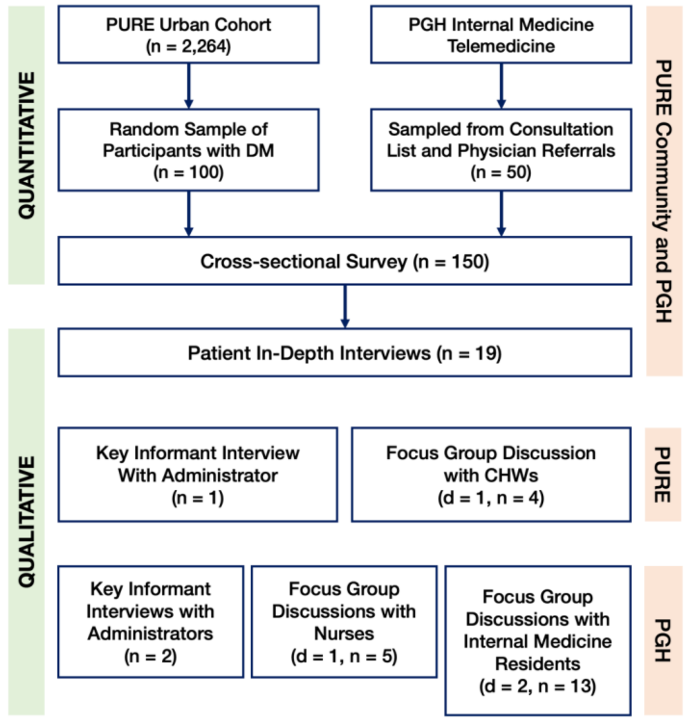
QUANTITATIVE
PURE Urban Cohort (n = 2,264)
PGH Internal Medicine Telemedicine
Random Sample of Participants with DM (n = 100)
Sampled from Consultation List and Physician Referrals (n = 50)
Cross-sectional Survey (n = 150)
PURE Community and PGH
QUALITATIVE
Patient In-Depth Interviews (n = 19)
Key Informant Interview With Administrator (n = 1)
Focus Group Discussion with CHWs (d = 1, n = 4)
PURE
Key Informant Interviews with Administrators (n = 2)
Focus Group Discussions with Nurses (d = 1, n = 5)
Focus Group Discussions with Internal Medicine Residents (d = 2, n = 13)
PGH

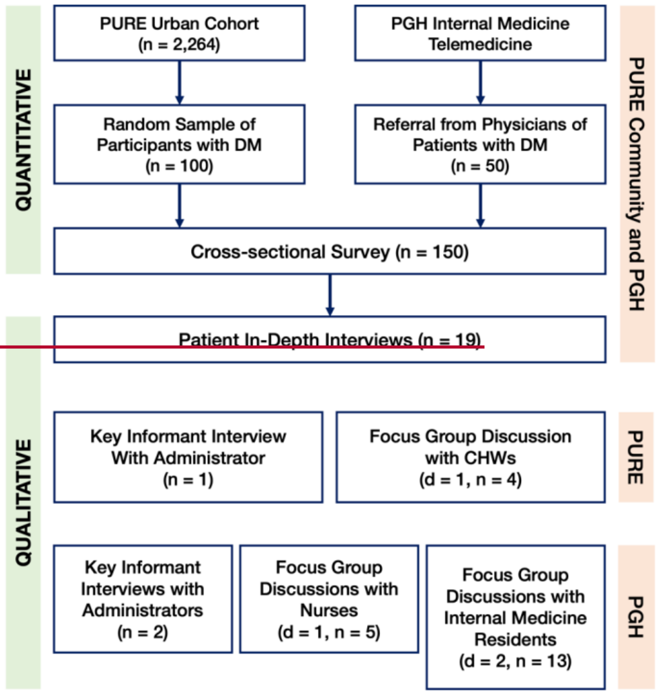

Fig 1. Study participant flow. CHW – community health worker, DM – diabetes mellitus, PGH – Philippine General Hospital, PURE – Prospective Urban and Rural Epidemiological Study; d – number of focus group discussions conducted, n – number of individual study participants included

Cross-sectional Survey Findings

Most of the participants enrolled in the study were middle-aged, female, married, and had completed at least secondary school education (Table 1). The median duration of diabetes diagnosis was 10 years, and the majority had at least one comorbidity, with hypertension being the most frequent.

Table 1. Demographic and clinical characteristics of study participants.

	N = 150 (%)
Age (years)	
Median [interquartile range]	62 [55.0, 68.0]
Age Groups	
19-39 years	3 (2.0)
40-59 years	54 (36.0)

60-69 years	58 (38.7)
70 years and above	35 (23.3)
Sex	
Female	106 (70.7)
Education	
Primary	13 (8.7)
High School	75 (50.0)
Vocational School	11 (7.3)
College/University	51 (34.0)
Marital Status	
Never married	14 (9.3)
Currently married	88 (58.7)
Separated/divorced/annulled	8 (5.3)
Widowed	40 (26.7)
Duration of Diabetes Mellitus (years)	
Median [interquartile range]	10.0 [8.0, 15.0]
Number of Any Comorbid Illness	
Median [interquartile range]	1 [0, 1.0]
Types of Comorbidities	
Hypertension	97 (64.7)
Heart Disease	14 (9.3)
Others	21 (14.0)

Before and during the pandemic, membership in any health insurance scheme and in PhilHealth, the ~~government-owned insurance system and~~ main implementing mechanism for universal health coverage [33], was consistently high (Table 2). However, the proportion of patients with any means of livelihood during the pandemic was significantly lower, and the proportion of patients below the PHP 5,000 monthly household income group during the pandemic were significantly higher than prior to the pandemic. There was no significant difference in household size between periods.

Table 2. Patient socioeconomic status before and during the pandemic.

	Pre-pandemic N (%)	During Pandemic N (%)	p value
Household Size			
≤ 5 members	99 (66.0)	102 (68.0)	0.623
> 5 members	51 (34.0)	48 (32.0)	
Presence of Means of Livelihood			
Yes	85 (56.7)	49 (32.7)	**<0.001**
Average Monthly Household Income (PHP)			
<5,000	9 (6.0)	26 (17.3)	**0.017**
5,000 – 20,000	89 (59.3)	76 (50.7)	
20,001 and above	26 (17.3)	20 (13.3)	
Unknown	26 (17.3)	28 (18.7)	
Membership to any Health Insurance Scheme			
Yes	124 (82.7)	123 (82.0)	0.948
PhilHealth membership			
Yes	115 (76.7)	117 (78.0)	0.617

There were significant differences in the diabetes care received by the patients between time periods (Table 3). Prior to the pandemic, most patients had been followed in the same healthcare facility for more than two years. However, during the pandemic, more than half of the participants reported having been followed in their healthcare facility for less than a year. Furthermore, 6.7% had to stop taking their medications completely. There was a significant reduction in glycemic control during the pandemic, and almost a quarter of participants did not have any fasting blood sugar (FBS) monitoring.

Table 3. Characteristics of diabetes care before and during the pandemic.

	Pre-pandemic N (%)	During Pandemic N (%)	p value
Duration of Follow-Up in the Healthcare Facility			
< 1 year	7 (4.7)	81 (54.0)	**<0.001**
1 to ≤ 2 years	23 (15.3)	24 (16.0)	
> 2 years	120 (80.0)	42 (28.0)	
Frequency of Medication Intake per Week			
Daily	129 (86.0)	119 (79.3)	**0.008**
Less than daily	21 (14.0)	21 (14.0)	
Stopped medications	0	10 (6.7)	
Controlled Glucose Level during Last Fasting Blood Sugar Testing			
Yes	78 (52.0)	59 (39.3)	**<0.001**
No	59 (39.3)	48 (32.0)	
Unknown	13 (8.7)	9 (6.0)	
No recent testing	0	34 (22.7)	

The proportion of participants who reported challenges in access to consultations, medications, and diagnostic testing during the pandemic increased by up to twice compared to pre-pandemic (Fig 2).

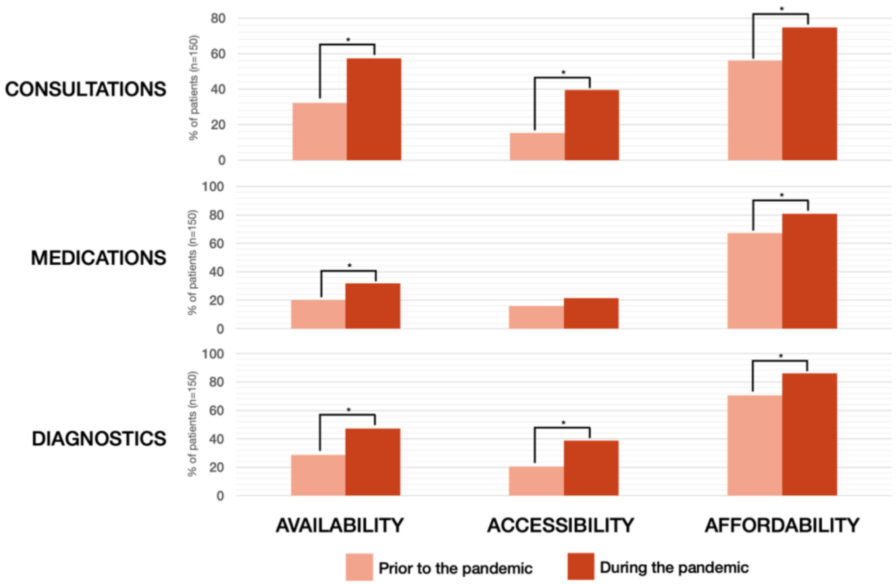

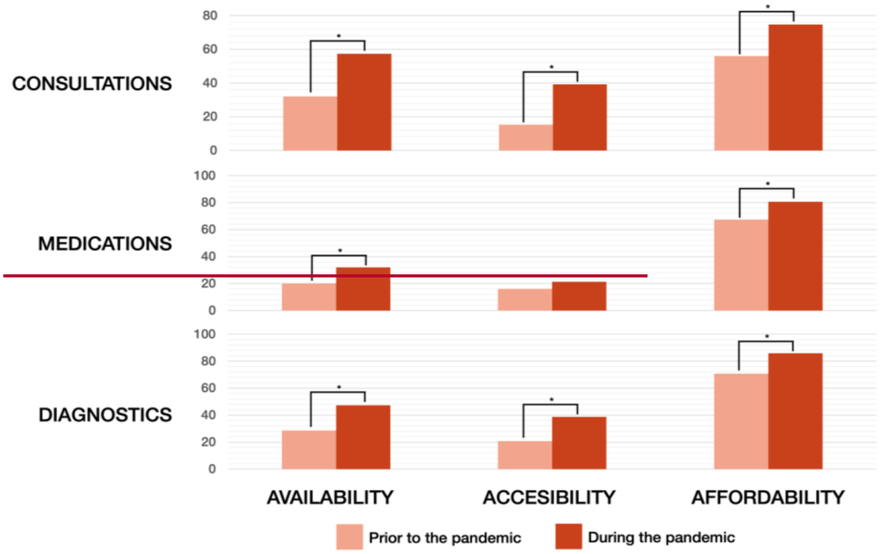

Fig 2. Patient-reported challenges in accessing diabetes care. The proportion of patients reporting challenges, defined as being problematic on categorization of Likert scales, in the respective component of diabetes care and domain of access is represented by the y-axis. Asterisks (*) indicate statistically significant differences ($p < 0.05$) between time periods. There was no correction for multiple comparisons.

Most participants also reported either foregoing necessities or borrowing money to afford consultations, medications, and diagnostic testing, or having no consultation/meds/tests during the pandemic (Table 4).

Table 4. Reported catastrophic health expenditure before and during the pandemic.

	Pre-pandemic N (%)	During Pandemic N (%)	p value
Foregoing Other Necessities or Borrowing Money to Afford Consultation			
Yes	36 (24.0)	40 (26.7)	**<0.001**
No	114 (76.0)	93 (62.0)	
No consult in the last 6 months	0	17 (11.3)	
Foregoing Other Necessities or Borrowing Money to Afford Medications			
Yes	40 (26.7)	50 (33.3)	**<0.001**
No	110 (73.3)	93 (62.0)	
No medication in the last 6 months	0	7 (4.7)	
Foregoing Other Necessities or Borrowing Money to Afford DM Laboratory Tests			
Yes	41 (27.3)	50 (33.3)	**<0.001**
No	109 (72.7)	89 (59.3)	
No testing in the last 6 months	0	11 (7.3)	

Qualitative Findings

The qualitative data revealed three overarching themes that influenced their access and the delivery of services for ~~DM~~ diabetes care during the pandemic – imposition of mandatory lockdowns, financial instability, and *covidization* (i.e., prioritization of COVID-19 above other societal problems) of the healthcare system (Table 5). These were consistent with our quantitative findings.

Table 5. Summary of qualitative findings.

Themes	Key Findings	Data Source
Imposition of mandatory lockdowns	1. Limited availability of facilities where patients could seek consultations and laboratory examinations affected patients' capacity to monitor their diabetes.	Patients
	2. Reduced social mobility and lack of public transportation options hindered access to health facilities which had impact on patients' capacity to monitor their diabetes and sustain medication.	
Financial instability	1. Losing sources of livelihood during the pandemic tremendously affected patients' capacity to afford consultations, laboratory exams, and medications.	Patients
	2. Trade-offs were present in the form of other competing household	

	expenses which led to suboptimal management for diabetes.	
	3. Limited capacity to afford medication resulted to different coping strategies such as shifting to herbal remedies, taking intentional medication breaks and utilizing social capital to borrow funds for medicine.	
Covidization of the healthcare system	1. The outpatient department, where diabetes was routinely managed, had to temporarily shut down their services for almost a year, effectively restricting access to non-COVID-19 healthcare needs.	Healthcare providers ~~and~~, administrators, and patients
	2. Even with telemedicine, a full assessment for DM patients was difficult to perform without the benefit of physical examination. Additionally, while telemedicine was able to revive provision of outpatient services including ~~DM~~ diabetes care, healthcare providers felt that many patients were still lost to follow-up due to disparate access to mobile devices.	
	3. Most of the human resources and health supplies were diverted to ~~ICU~~ critical care units to prioritize those with COVID-19. This resulted in delays and suboptimal treatment for admitted patients with complications or severe symptoms from uncontrolled glucose levels.	

Imposition of mandatory lockdowns

The recurrent lockdowns ~~which were part of the social measures to curb the transmission of the virus~~ resulted in the suspension of face-to-face consultations and laboratory examinations. Many of the patients in the interview reported having no consultations and examinations done for at least six months, especially during the first two years of the COVID-19 pandemic. Reduced monitoring resulted in uncertainty regarding their diabetes status which they felt compelled to accept. As shared by patient **PG4 (55 years old, female)**:

When the pandemic started, all those necessary check-ups were totally gone. You know I didn't really know how my diabetes was doing because I wasn't able to go back (to PGH OPD). Even though I know that check-ups and laboratory exams are important, I can't just go out because it was prohibited. It was very difficult during the pandemic.

~~As part of the mandated social restrictions, public~~Public transportation became limited as well. ~~Traveling measures~~Rules on travel were stringent within Metro Manila and even more so for those coming from outside of the region. Despite the eventual ease on heavy lockdowns, travel constraints also had negative impact in accessing facilities for ~~DM~~ diabetes care.

The restrictions on social mobility coupled with the fear of contracting COVID-19 also impeded some of the patients' access to ~~DM~~ diabetes care ~~services~~. This was revealed in the account of patient **PG3 (59 years old, female)**:

> *For almost two years, I didn't have any consultations and laboratory exams, but I was taking my medicine continuously. It was the only thing I could do. We were not allowed to go out especially us the elderlies. But I am also afraid to go out even when the most restrictive lockdown was lifted. I still decided not to get any laboratory exams because I don't know, I might get COVID in the diagnostic facility.*

Different experiences regarding access to medications were reported. Some recounted not having any challenges with getting their diabetes medications because pharmacies were still accessible ~~to many~~ even during lockdowns. While for others, the imposition of granular (i.e., small-scale) lockdowns in certain households ~~where active cases of COVID-19 were being monitored~~ precluded any social movement and imposed barriers to medication adherence for certain periods of time.

Financial instability

Many of the patients reported losing their source of livelihood during the pandemic. Such experience was not ~~only~~ exclusive to the patients themselves but was also a problem for their family members and relatives who often helped ~~financing~~ finance ~~health~~ diabetes care expenditures ~~for DM care~~. Financial instability greatly affected patients' capacity to afford consultations, laboratory exams, and medications. This is exemplified in the succeeding quote:

> **PU10 (50 years old, male):** *My last check-up and laboratory was back in 2019. When the pandemic started, the company I worked for had to lay-off some employees, and I was included. I had a Medicard then* (health insurance from work) *which I used to finance my health expenses for diabetes. Right now I am just continuing the Metformin… I'm still not sure when can I have a check-up again because money is an issue. Also, right now you can't just go to the facilities because they require swab tests, that's another thing to be concerned about because you to pay for it.*

Meeting the financial demands for ~~DM~~ diabetes care resulted in trade-offs. Health expenses competed with other household expenses leading to suboptimal self-management for diabetes as shown in the illustrative quotes below:

> **PU16 (73 years old, female):** *To tell you the truth money was really an issue. My two sons who are living with me lost their jobs, but I am very fortunate that my grandchildren are now paying for my laboratory and*

medicine. However, money is still tight. Sometimes I buy medicine that's insufficient for a week because we had to pay for water and electricity bills.

PG7 (45 years old, male): *It was difficult financially. I had problems securing money for my laboratory exams and medications. My work days weren't as frequent as before and my wife lost her job. Plus, we still had other bills to pay.*

Difficulties ~~with~~ in purchasing ~~medicine~~ medications were met with different coping strategies intended to manage diabetes within the best of patients' capabilities. These actions include shifting to herbal remedies and taking intentional medication breaks. Some also utilized their social capital ~~in the form of~~by borrowing money from friends and relatives~~ to purchase medication~~. The FGD with CHWs from the PURE community also revealed they had to ~~make adjustments~~adapt to meet the needs of DM patients within their catchment areas, who mainly relied on free diabetes medication from the local health centers (e.g., house-to-house visit to personally deliver medication to DM patients).

Covidization of healthcare system

The *covidization* of healthcare has been ~~noted elsewhere~~previously described to illustrate how pandemic-related policies dominated and restructured health service delivery and health research [34–36]. It alludes to the outpouring of resources and attention solely to COVID-19 thereby deprioritizing other serious and pressing health concerns such as the management for chronic conditions.

As revealed in the FGDs with HCWs and administrators, the unavailability of non-COVID healthcare services was ~~also~~ a major dilemma for continued ~~DM~~ diabetes care. Resident doctors from PGH were consistent in reporting that many of their DM patients were unchecked or lost to follow-up during the first year of the pandemic primarily because the OPD had to discontinue services. Many ~~of them~~ had no contact with their usual DM patients. To some extent t~~T~~he introduction of telemedicine ~~after a year to some extent~~ reinstated the delivery of care to OPD patients including those with diabetes. ~~Nevertheless~~However, ~~the resident doctors and nurses~~ healthcare workers still estimated that they were seeing far fewer DM patients than they used to pre-pandemic.

~~With~~ Despite the influx of DM patients who were using teleconsultations, many were still unable to monitor their glucose levels ~~citing~~ due to social restrictions and lack of funds~~ as the main reasons~~. ~~Moreover, r~~Resident doctors agreed that providing medication instructions ~~regarding medication~~ was one of the ~~big~~ major challenges ~~with~~ in using telemedicine. Without the benefit of the physical examination, it was ~~harder~~ more difficult to fully assess the diabetes condition of many ~~DM~~ patients, especially those who had gone without laboratory testing or had no access to ~~monitor their~~ capillary blood glucose ~~levels~~monitoring. ~~Additionally, i~~Instructing patients ~~regarding~~ on insulin use was another struggle for both healthcare providers and ~~users~~patients. Physicians were seeing much more hesitancy among patients towards starting insulin after receiving initial instructions through teleconsultations.

Telemedicine became instrumental in bridging access ~~and availability of services to non-COVID cases including diabetes~~to diabetes care, but ~~. However,~~ it came with its own challenges. On a micro level, this included inadequate funding to support the sustained operation of telemedicine within the hospital according to administrators. Inequalities regarding access to mobile devices and disparities on technological literacy were also common challenges identified. Hence, while telemedicine was able to revive provision of outpatient services ~~including DM care, HCWs~~, healthcare workers and administrators felt that many patients were still lost to follow-up ~~as a result of~~due to COVID-19 restrictions.

The FGDs also revealed that most of the human resources and health supplies were diverted to ~~ICU~~ critical care units to prioritize ~~those with~~ COVID-19 patients. This resulted in delays and suboptimal treatment for in~~admitted~~ patients with complications or severe symptoms from uncontrolled glucose levels.

Many patients were also forced to move from one health facility to another to find one that would cater to their non-COVID related concerns. This scenario is described more vividly by patient **PU14 (female, 56 years old)** when she needed emergency care:

> *"There was a time when I was very ill, my family had to send me to a hospital. Turned out my blood sugar was very low. We went to three different public hospitals first, and they all turned us down because they were focused on COVID. We had no choice but to find an alternative that was a private facility where I can get accommodated but we had to pay. That wasn't a pleasant experience."*

Meanwhile, the community doctor and ~~other CHWs~~staff members in one of the PURE study sites mentioned having insufficient supply of medication for diabetes and hypertension and further delays in restocking these medicines because of the periodic lockdowns. Additionally, ~~the services for DM became~~diabetes care services were limited to dispensing medication. ~~They suspended their~~ FBS monitoring service was suspended, and ~~because of the social restrictions, lack of manpower, and equipment. T~~the health center staff also ~~had to take~~took on new ~~roles and~~COVID-19-related responsibilities ~~which were more focused on COVID-19~~ such as contact tracing, rapid testing, vaccination, and ~~even~~ facilitating of cremation services.

Triangulating Findings

Overall, we found a strong congruence between the quantitative and qualitative findings. The reported limited availability of ~~DM~~ diabetes care during the pandemic were largely attributed to the *covidization* of the healthcare system. This was especially felt in Metro Manila, which was the region most severely affected by COVID-19 in the country. The reordering of priorities of health facilities and their human resources to COVID-19 activities may have led some patients to discontinue follow-up with their usual ~~DM~~diabetes-care provider and

seek care in other facilities. These changes may have contributed to the reported increase in the proportion of patients with <1 year follow-up in their most current healthcare facility.

Similarly, the limitations in the accessibility of consultations and laboratory examinations emerged from the imposition of mandatory lockdowns. In the cross-sectional survey, accessibility to medications did not significantly change during the pandemic~~, which was likely due to the non-closure of pharmacies~~. However, as shown by the interviews, some patients who experienced granular lockdowns could not reach drug stores, limiting medication adherence. This was reflected in the reduction in daily medication intake of participants during the pandemic.

Finally, the financial instability experienced by patients and their families meant that it was often difficult to afford appropriate ~~DM~~ diabetes care. Due to loss of livelihood by patients and/or their caregivers, trade-offs including self-management of diabetes were made, often without professional guidance.

Discussion

~~In this study, w~~We aimed to describe the impact of the COVID-19 pandemic on diabetes care in Metro Manila, primarily from the vantage point of patients, as corroborated by ~~HCWs~~ healthcare workers and ~~health facility~~ administrators.

We found adverse changes in the socioeconomic status and diabetes care of patients during the pandemic, most evident in ~~the~~ greater proportions of individuals in the lowest income group (i.e., PHP 5,000), which was well below the poverty threshold of PHP 12,030 per month for a family of five [37]. Similarly, that a greater proportion of patients had less than a year of follow up in their current facility during the pandemic suggests that they may have felt compelled to change their facility for consultations. The alarming reduction in the number of patients able to perform FBS monitoring also shows disruptions in a key component of diabetes care [17].

The ~~existing~~ challenges encountered by patients in accessing diabetes care prior to the pandemic were aggravated during the pandemic. Across all domains and areas of diabetes care, more patients reported challenges ~~increased~~ during the pandemic, but the accessibility of diabetes medications had not significantly changed, likely due to the continued operations of pharmacies ~~despite~~ during lockdowns. Despite high enrollment (>75%) into PhilHealth and other health insurance schemes, and service provision being free at point of care in PGH and ~~local~~ health centers, most of the patients ~~also~~ found challenges in the affordability of consultations, diagnostics, and medications~~ even pre-pandemic~~. These ~~responses~~ difficulties were reflected in our finding that about a quarter of patients experienced catastrophic health expenditures even prior to 2020. ~~These difficulties worsened in the pandemic, with~~The affordability of diagnostics ~~being the most severely affected area of DM care~~during the pandemic was especially problematic. Our findings echo the well-documented dissociation between PhilHealth

enrollment and catastrophic health expenditure [38–40]~~ and highlight the magnitude of work needed to ensure that universal health care becomes a reality in the country~~.

~~The patients identified the imposition of lockdowns, financial instability, and the *covidization* of the healthcare system as contributors to the identified challenges. In response to the difficulties encountered, some patients coped through DM self-management. HCWs and administrators alike deemed the use of telemedicine as an important tool to reach patients but suspected that many more patients did not have easy access to telemedicine and were lost to follow up during the pandemic. They also identified medication supply limitations and higher prioritization of the healthcare system for COVID-19 public health activities as barriers to non-COVID-19 service delivery at the community level.~~

Studies on the collateral adverse impact of COVID-19 on healthcare ~~and ultimately on health~~ in the Philippines are limited. ~~A systematic literature review of policies and qualitative survey of physicians and administrators in early 2021 assessed the impact of COVID-19 on the health system of the country and found that community quarantines and border restrictions universally impacted health service access and delivery, with greater cascading effects among those needing specialist care. [9]~~ A~~nother~~ qualitative study of public health workers, university staff, and administrators explored the impact the pandemic on local health systems and higher education institutions in ~~Luzon, Visayas, and Mindanao~~the country. ~~That study found that r~~Routine health programs were found to be inaccessible due to changing ~~health program~~ priorities and lack of students to augment the health workforce [41]. ~~[41]~~ The results ~~of these Philippine-based studies~~ were consistent with previous research in other less-resourced contexts [42,43]. ~~[42,43]~~ Despite suggesting patient hesitancy to access health facilities as a challenge, these studies ~~only included the perspectives of healthcare workers, administrators, and general community members~~did not include the perspectives of healthcare users.

Our study is the first to ~~include and~~ highlight the patient perspective in assessing the indirect effects of COVID-19 on other health programs in the country. We broke down health service access and delivery ~~down~~ into availability, accessibility, and affordability of consultations, diagnostics, and medications to more readily identify areas that can be prioritized for improvement. The use of mixed methods allowed us to better characterize the patient experience beyond the constraints imposed by our deconstruction and conceptualization of "access". Furthermore, the perspectives of ~~HCWs~~ healthcare workers and administrators helped identify challenges in the local health centers and those associated with the use of telemedicine in the hospital.

Our work adds evidence from the Philippines, which ~~experienced~~ implemented one of the most restrictive responses to the COVID-19 pandemic, about the indirect impact of global ~~catastrophes~~ pandemics on health. The probability of another extreme novel pandemic occurring within a lifetime is high [44], and as governments and health systems reckon with the aftermath of

COVID-19, our work echoes the need to integrate NCD care into pandemic preparedness efforts. Our work also shows how the government's securitization of health and its response to the pandemic may have exacerbated already existing health inequities and highlights the importance of ~~It is important to~~ prioritizing~~e~~ health financing strategies in the country to reduce catastrophic health expenditure and to make healthcare truly accessible to all.

Our study has several limitations. First, the relatively small sample size used in the cross-sectional study was only powered to estimate the proportion of the population not receiving DM care and not necessarily the difference in outcomes between two time points. Furthermore, we did not adjust our statistical tests for multiple comparisons. However, even with the limited sample size, the results of the quantitative component consistently suggest generally worse conditions during the COVID-19 pandemic, across different access domains and areas of care.

Second, the catchment population of PGH and the urban location of the community cohort may limit the generalizability of our results to other areas in the Philippines. On the one hand, the longer and stricter lockdowns in Metro Manila would seem to impose greater accessibility issues in the region compared to those outside. On the other hand, the provinces and geographically isolated and disadvantaged areas also experienced lockdowns and resource constraints, and patients may have had even greater accessibility issues compared to those in Metro Manila.

Third, our sampling strategy may have introduced selection bias. By including PGH OPD patients and community-dwelling participants already involved in a different study, ~~our~~ the study participants were more likely to already have had better access to healthcare and other positive health-reinforcing behaviors than the average patient with DM. Furthermore, because we excluded patients managed by the diabetes specialty clinic who we assumed to have greater DM care needs, our results are more applicable to assess issues in the primary care setting.

~~Finally~~Fourth, inherent in our survey questionnaire design is the potential for recall bias, especially regarding the circumstances prior to the pandemic, and recency bias concerning the events during the pandemic. Social desirability bias may have also discouraged participants to admit borrowing money to afford consultation, medications, and laboratory tests; thus, this may underestimate actual catastrophic health expenditure.

Lastly, we iteratively implemented the explanatory, sequential typology such that the cross-sectional study results were not used to inform the construction of the qualitative study guides. Rather, we used the quantitative results to partly inform the analysis of the qualitative portions of the study. We deemed this appropriate in validating our key findings from the patient perspective as corroborated by insights from healthcare workers and administrations.

Formatted: Font: 12 pt

Formatted: Font: 12 pt

Conclusion

The imposition of lockdowns, *covidization* of the healthcare system, and financial instability contributed to the reduced availability, accessibility, and affordability of diabetes care-related consultations, laboratory tests, and medications during the COVID-19 pandemic. These combined effects of the pandemic prompted some patients to self-manage their condition, often without professional guidance. Telemedicine was viewed by healthcare workers and administrators as a promising yet incomplete bridge to reach both new patients and patients lost to follow-up. Our findings highlight the need to integrate NCD care in pandemic preparedness efforts. Our study supports calls to prioritize health financing reforms in the Philippines to prevent catastrophic health expenditure, especially for primary healthcare conditions necessitating routine outpatient care. The reforms are urgently needed now and in preparation for the next pandemic.

Acknowledgement

We thank Dr. Jhoanna Rose Velasquez for contributing to the conceptualization and ethics approval application for the study. We acknowledge the assistance provided by Chareece Mae Zamora-Pudol, Louie Pudol, and Rachel Cabrega in conducting the cross-sectional study. We also acknowledge the assistance provided by Eunice Mallari in conducting and analyzing the qualitative study.

References

1. World Health Organization. Maintaining essential health services: operational guidance for the COVID-19 context: interim guidance, 25 March 2020 [Internet]. Geneva: World Health Organization; 2020 Mar [cited 2023 Feb 27]. Available from: https://apps.who.int/iris/handle/10665/331561

2. World Health Organization. Maintaining essential health services: operational guidance for the COVID-19 context: interim guidance, 1 June 2020 [Internet]. World Health Organization; 2020 [cited 2023 Feb 27]. Report No.: WHO/2019-nCoV/essential_health_services/2020.2. Available from: https://apps.who.int/iris/handle/10665/332240

3. World Health Organization. Regional Office for South-East Asia. Integration of NCD care in emergency response and preparedness [Internet]. World Health Organization. Regional Office for South-East Asia; 2018 [cited 2023 Jul 20]. Available from: https://apps.who.int/iris/handle/10665/272964

4. World Health Organization. The impact of the COVID-19 pandemic on noncommunicable disease resources and services: results of a rapid

assessment [Internet]. Geneva: World Health Organization; 2020 [cited 2022 Dec 10]. Available from: https://www.who.int/publications-detail-redirect/9789240010291

5. Gadsden T, Downey LE, Vilas VDR, Peiris D, Jan S. The impact of COVID-19 on essential health service provision for noncommunicable diseases in the South-East Asia region: A systematic review. The Lancet Regional Health - Southeast Asia [Internet]. 2022 Jun 1 [cited 2022 Dec 10];1. Available from: https://www.thelancet.com/journals/lansea/article/PIIS2772-3682(22)00010-5/fulltext

6. Nikoloski Z, Alqunaibet AM, Alfawaz RA, Almudarra SS, Herbst CH, El-Saharty S, et al. Covid-19 and non-communicable diseases: evidence from a systematic literature review. BMC Public Health. 2021 Jun 5;21(1):1068.

7. Hapal K. The Philippines' COVID-19 Response: Securitising the Pandemic and Disciplining the Pasaway. Journal of Current Southeast Asian Affairs. 2021 Aug 1;40(2):224–44.

8. Paras AY. An Evolutionary Institutionalist Explanation of the Philippines' Militaristic COVID-19 Approach. Soc. 2022 Aug 1;59(4):359–65.

9. Bayani DB, Tan SG. Health Systems Impact of COVID-19 in the Philippines [Internet]. [cited 2023 Feb 8]. Available from: https://www.cgdev.org/publication/health-systems-impact-covid-19-philippines

10. Ulep VG, Paterno A, Uy J, Van VS, Casas L, Tan J. The Impact of the COVID-19 Pandemic on Social Health Insurance Claims for High-Burden Diseases in the Philippines [Internet]. Washington DC: Center for Global Development; 2021 May [cited 2023 Jul 20]. Available from: https://www.cgdev.org/publication/impact-covid-19-pandemic-social-health-insurance-claims-high-burden-diseases-philippines

11. Uy J, Van VTS, Ulep VG, Bayani DB, Walker D. The Impact of COVID-19 on Hospital Admissions for Twelve High-Burden Diseases and Five Common Procedures in the Philippines: A National Health Insurance Database Study 2019-2020. The Lancet Regional Health – Western Pacific [Internet]. 2022 Jan 1 [cited 2023 Jul 20];18. Available from: https://www.thelancet.com/journals/lanwpc/article/PIIS2666-6065(21)00219-4/fulltext

12. Levesque JF, Harris MF, Russell G. Patient-centred access to health care: conceptualising access at the interface of health systems and populations. International Journal for Equity in Health. 2013 Mar 11;12(1):18.

13. An J, Le QA, Dang T. Association between different types of comorbidity and disease burden in patients with diabetes. J Diabetes. 2019 Jan;11(1):65–74.

14. Nolte E, Bain C, McKee M. Diabetes as a Tracer Condition in International Benchmarking of Health Systems. Diabetes Care. 2006 May 1;29(5):1007–11.

15. Lindström K, Berg L, Rylander B, Hagman A, Olsson L, Bengtsson C. A model for quality assessment in primary health care using the tracer condition technique with insulin treated diabetes as one of the tracers. Scandinavian Journal of Primary Health Care. 1997 Jan 1;15(2):92–6.

16. Chudasama YV, Gillies CL, Zaccardi F, Coles B, Davies MJ, Seidu S, et al. Impact of COVID-19 on routine care for chronic diseases: A global survey of views from healthcare professionals. Diabetes & Metabolic Syndrome: Clinical Research & Reviews. 2020 Sep 1;14(5):965–7.

17. American Diabetes Association. Standards of Medical Care in Diabetes—2022 Abridged for Primary Care Providers. Clinical Diabetes. 2022 Jan 1;40(1):10–38.

18. Mohseni M, Ahmadi S, Azami-Aghdash S, Isfahani HM, Moosavi A, Fardid M, et al. Challenges of routine diabetes care during COVID-19 era: A systematic search and narrative review. Primary Care Diabetes. 2021 Dec 1;15(6):918–22.

19. PHRI.ICT W. PURE – Prospective Urban and Rural Epidemiological Study [Internet]. [cited 2022 Dec 11]. Available from: https://www2.phri.ca/pure/

20. Dayrit M, Lagrada L, Picazo O, Pons M, Villaverde M. The Philippines health system review. Health Systems in Transition [Internet]. 2018 [cited 2022 Dec 11];8(2). Available from: https://apps.who.int/iris/handle/10665/274579

21. Microsoft. Microsoft Excel Spreadsheet Software | Microsoft 365 [Internet]. [cited 2022 Apr 19]. Available from: https://www.microsoft.com/en-ww/microsoft-365/excel

22. Department of Science and Technology - Food and Nutrition Research Institute (DOST-FNRI) M. Philippine Nutrition Facts and Figures - 2018 Expanded National Nutrition Survey [Internet]. Manila: DOST-FNRI; 2020 [cited 2023 Feb 8]. Available from: http://enutrition.fnri.dost.gov.ph/site/uploads/2018_ENNS_Facts_and_Figures.pdf

23. Singh ND and M. Sample Size Calculator for Estimating a Proportion [Internet]. [cited 2023 Feb 8]. Available from: https://www.statulator.com/SampleSize/ss1P.html

24. Dworkin SL. Sample Size Policy for Qualitative Studies Using In-Depth Interviews. Arch Sex Behav. 2012 Dec 1;41(6):1319–20.

25. Vasileiou K, Barnett J, Thorpe S, Young T. Characterising and justifying sample size sufficiency in interview-based studies: systematic analysis of qualitative health research over a 15-year period. BMC Medical Research Methodology. 2018 Nov 21;18(1):148.

26. Faulkner SL, Trotter SP. Data Saturation. In: The International Encyclopedia of Communication Research Methods [Internet]. John Wiley & Sons, Ltd; 2017 [cited 2023 Feb 7]. p. 1–2. Available from: https://onlinelibrary.wiley.com/doi/abs/10.1002/9781118901731.iecrm0060

27. Guest G, Namey E, Chen M. A simple method to assess and report thematic saturation in qualitative research. PLOS ONE. 2020 May 5;15(5):e0232076.

28. RStudio Team. RStudio: Integrated Development for R [Internet]. Boston, MA: RStudio, PBC; 2021 [cited 2022 Apr 5]. (Ghost Orchid). Available from: https://www.rstudio.com/

29. Onwuegbuzie AJ, Leech NL. On Becoming a Pragmatic Researcher: The Importance of Combining Quantitative and Qualitative Research Methodologies. International Journal of Social Research Methodology. 2005 Dec 1;8(5):375–87.

30. Lee SYD, Iott B, Banaszak-Holl J, Shih SF, Raj M, Johnson KE, et al. Application of Mixed Methods in Health Services Management Research: A Systematic Review. Med Care Res Rev. 2022 Jun 1;79(3):331–44.

31. von Elm E, Altman DG, Egger M, Pocock SJ, Gøtzsche PC, Vandenbroucke JP, et al. The Strengthening the Reporting of Observational Studies in Epidemiology (STROBE) statement: guidelines for reporting observational studies. PLoS Med. 2007 Oct 16;4(10):e296.

32. O'Brien BC, Harris IB, Beckman TJ, Reed DA, Cook DA. Standards for reporting qualitative research: a synthesis of recommendations. Acad Med. 2014 Sep;89(9):1245–51.

33. Congress of the Philippines. Republic Act No. 7875 | AN ACT INSTITUTING A NATIONAL HEALTH INSURANCE PROGRAM FOR ALL FILIPINOS AND ESTABLISHING THE PHILIPPINE HEALTH INSURANCE CORPORATION FOR THE PURPOSE [Internet]. 7875. Available from: https://www.philhealth.gov.ph/about_us/ra7875.pdf

34. Lasco G, San Pedro J. The 'covidization' of health care | Inquirer Opinion. Inquirer [Internet]. 2020 Jun 7 [cited 2023 Feb 28]; Available from: https://opinion.inquirer.net/130544/the-covidization-of-health-care

35. Pai M. Covidization of research: what are the risks? Nat Med. 2020 Aug;26(8):1159–1159.

36. Alcalde J. Covidization Of Healthcare Leaves Other Diseases Untreated. 2021 Jan 5 [cited 2023 Feb 28]; Available from: https://worldcrunch.com/st/static_newsletter_page

37. Philippine Statistics Authority. Proportion of Poor Filipinos was Recorded at 18.1 Percent in 2021. 2022 Aug 15 [cited 2023 Feb 28]; Available from: https://psa.gov.ph/sites/default/files/3-%28ons-cleared%29_Rev_Press%20Release%20%28for%20posting%29_2021FY%2C%2011Aug2022_wFooter_wag_ONSF-signed.pdf

38. Lasco G, Yu VG, David CC. The Lived Realities of Health Financing: A Qualitative Exploration of Catastrophic Health Expenditure in the Philippines. Acta Medica Philippina [Internet]. 2022 Jun 29 [cited 2023 Feb 8];56(11). Available from: https://actamedicaphilippina.upm.edu.ph/index.php/acta/article/view/2389

39. Querri A, Ohkado A, Kawatsu L, Remonte MA, Medina A, Garfin AMC. The challenges of the Philippines' social health insurance programme in the era of Universal Health Coverage. Public Health Action. 2018 Dec 12;8(4):175.

40. Bredenkamp C, Buisman LR. Financial protection from health spending in the Philippines: policies and progress. Health Policy and Planning. 2016 Sep 1;31(7):919–27.

41. Maravilla J, Catiwa J, Guariño R, Yap JF, Pagatpatan C, Orolfo DD, et al. Exploring indirect impacts of COVID-19 on local health systems from the perspectives of health workers and higher education stakeholders in the Philippines using a phenomenological approach. The Lancet Regional Health – Western Pacific [Internet]. 2023 Jan 1 [cited 2023 Feb 8];30. Available from: https://www.thelancet.com/journals/lanwpc/article/PIIS2666-6065(22)00200-0/fulltext#seccesectitle0020

42. Singh DR, Sunuwar DR, Shah SK, Karki K, Sah LK, Adhikari B, et al. Impact of COVID-19 on health services utilization in Province-2 of Nepal: a qualitative study among community members and stakeholders. BMC Health Services Research. 2021 Feb 24;21(1):174.

43. Khan MS, Rego S, Rajal JB, Bond V, Fatima RK, Isani AK, et al. Mitigating the impact of COVID-19 on tuberculosis and HIV services: A cross-sectional survey of 669 health professionals in 64 low and middle-income countries. PLoS One. 2021;16(2):e0244936.

44. Marani M, Katul GG, Pan WK, Parolari AJ. Intensity and frequency of extreme novel epidemics. Proceedings of the National Academy of Sciences. 2021 Aug 31;118(35):e2105482118.

8 August 2023

Prof. Catherine Kyobutungi
Prof. Madhukar Pai
Editors-in-Chief
PLOS Global Public Health

Through: **Nicola L. Hawley**
Academic Editor
PLOS Global Public Health

We thank the editors and reviewers for taking great interest in our work and for providing important and actionable feedback that we hope will help improve our manuscript and make our article meet the current journal standards.

In the following table, we respond to the comments and highlight where we implemented the changes in the revised submission.

Comments	Authors' Responses
**Journal Requirements**	
In the ethics statement in the Methods, you have specified that verbal consent was obtained. Please provide additional details regarding how this consent was documented and witnessed, and state whether this was approved by the IRB.	We provided additional details of the verbal consent process for both quantitative and qualitative components (see **pg 6, ln 6-11 and pg 8, ln 10-11**). The consent procedures are part of the study protocol, approved by the IRB, and we highlighted it in the ethics statement (see **pg 9, ln 24**).
We ask that a manuscript source file is provided at Revision. Please upload your manuscript file as a .doc, .docx, .rtf or .tex.	We have uploaded the manuscript file as .DOCX.
Please provide separate figure files in .tif or .eps format.	We have uploaded Figures 1 and 2 using .TIFF in accordance with journal guidelines.
**Reviewer #1**	
Please define 'covidization' earlier in the paper. Presently it is explained well on page 15, lines 44-45; however, since this phrase is still fairly new to the literature and field, it is recommended this definition be placed earlier in the paper upon the first use of the term.	We included a short description of *covidization* on **pg 14, ln 3-4** upon first use of the term. We purposefully refrained from introducing the concept in earlier sections (e.g., Introduction) because the concept and term emerged as part of the qualitative study, especially from healthcare worker FGDs and administrator interviews. Instead, we provided additional description of the government response to the pandemic in the Introduction (**pg 3, ln 6-19**) to give more context to the phenomenon.
To clarify the explanatory sequential mixed methods design, it would be useful to emphasize that the quantitative data was collected/analyzed first and informed the collection and analysis of the qualitative portion. A figure (Similar to the Figure 1 diagram included would be useful in demonstrating/illustrating the analytic process).	We clarified the description of our analyses, with close attention to how we used the explanatory sequential typology, on **pg 9, ln 1-14**.
As written, the paper leads one to presume that much of the analyses were conducted at very similar time points (this presumption is derived from the statement that participants who completed questionnaires were asked presumably directly afterwards if they wanted to participate in an interview). A strict mixed methods researcher may argue this could fall more into the realm of a convergent parallel design (especially with the emphasis	Thank you for recognizing our conceptualization of the explanatory approach. As suggested, we have reflected the non-classical application of the typology in the limitation on **pg 20, ln 33-39**.

on triangulation of the results). However, there is often flexibility, and as you state, the qualitative portions (including IDIs, KIIs, and FGDs), were used to expand, and explain the quantitative findings as per an explanatory approach. However, the researchers may want to briefly mention this as one minor limitation (that the quantitative results may not have been used sequentially to inform the construction/analysis of the qualitative portion).	
For example, was there a particular rationale for why only n = 1 of the key informant interviews were conducted from the PURE study?	We provided additional information on the considerations in the recruitment for qualitative component on **pg 7, ln 36 to pg 8, ln 11**. We considered the structure of the facility and the staff availability. In this particular instance, there as only one concerned administrator in the health facility serving the PURE study urban community cohort.
Page 4, lines 24-25; it is unclear what the (Barangays 178 to 186) is referring to.	The barangay is to the smallest administrative unit in the Philippines. We rephrased to statement to clarify this on **pg 4, ln 25-26**.
What was the rationale for n = 30 as an ideal sample size?	The ideal sample size of 30 was mentioned based on the proposal of earlier qualitative researchers which we have cited. We clarified this on **pg 7, ln 38**.
What were the screening criteria for inclusion for the IDIs?	Patients were screened for inclusion into IDIs primarily based on their willingness/consent and availability. However, we aimed to provide maximum variation according to age distribution, sex difference, and geographic location. We clarified this on **pg 7, ln 35-37**.
Can greater information/clarity be provided about the data availability of the PURE study? How did the researchers access these data to sample participants?	One of the study participants was also an investigator of PURE study. We sought permission from the PURE study team to access the list of potentially eligible participants in the urban community cohort. The line list of participants was made available to the mixed method study investigators. We clarified this on **pg 5, ln 33-35**.
Page 6: lines 35 – 40; what was the basis for assuming 50% of the individuals would report not having access to diabetes care during the pandemic?	Because we did not have any reliable estimate of population level proportion of individuals not having access to care during the pandemic, we used a conservative estimate of 50%, which would result in larger minimum sample size needed.
Was the assumption of 8% of the cohort having diabetes derived from the included citation?	The assumption of 8% prevalence was based on the national prevalence of high fasting blood glucose levels among Filipino adults residing in urban areas, which was derived from the citation (Expanded National Nutritional Survey 2018). We clarified this on **pg 8, ln 2-5**.
The researchers may want to consider including the interview and/or focus group agendas as supplementary material.	We have included the guides in the submission as **S2 Appendix**.
Page 7, lines 27-28: Since multiple criteria can be used to determine saturation, it may be useful to include what criteria was used here.	We specified the criteria we used to determine saturation on **pg 7, ln 39-40**.
Page 7, lines 40-41; It may be useful to see what the values represented. For example, if 10 is very easy, one can presume that 9 may be 'easy', which leads to questions as to why it would have been defined as problematic.	In the actual questionnaire, we did not provide descriptors between 1 and 10. However, we similarly presumed that two-point difference would be considered problematic. In the original manuscript we described *any value less than 9 to be problematic*. To better clarify the categorization we made, we changed

	it to *any value less than or equal to 8 to be problematic* (**pg 8, ln 19**).
While clearly defined in the manuscript, the number of acronyms used is overwhelming in some sections. It is recommended that alternative phrasing be used.	We have made some adjustments throughout the manuscript to address this (e.g., minimizing use of "DM", "HCWs", and "CHWs" and rephrasing statements to minimize acronyms).
It may help the readability and accessibility of this type of study to a wider audience if the researchers could reorganize the results and discussion sections to be a bit more concise (there is some repetition about covidization especially within these sections).	We have made some adjustments throughout the manuscript to address this. We moved some parts of the discussion to introduction (see **pg 3 ln 10-18**) and omitted repetitive paragraphs (e.g., we removed **pg 18 ln 28-36 of the original manuscript submission**). We also simplified some of the paragraphs in the results and discussion sections.
**Reviewer #2**	
Introduction: more context into how COVID impacted/disrupted health services in the Philippines in particular; there is great data from worldwide effects but want more local information, particularly about diabetes (and other NCDs)	While the evidence on this is sparse, we have restructured the introduction to better highlight these points (see **pg 2, ln 34 to pg 3, ln 19**).
It is unclear what convenience sampling refers to and why participants were recruited in this method. Is the population recruited from PGH OPD equal to those recruited from the PURE cohort? Are these populations the same? What was the follow up on patients ?	The convenience sampling mentioned in the recruitment from PGH OPD primarily refers to the use of line list of patients who were scheduled for follow-up telemedicine consult and to the referral of resident doctors of their patients to the study investigators. 100 of patients were recruited from PURE cohort, and 50 were recruited from PGH OPD. Because of the prevailing restrictions at the time, we put a strong emphasis on the availability of the potential participants and study personnel to collect data, and this was reflected in the recruitment design. The population differed in where they access healthcare, and there was no follow-up for patients recruited in the cross-sectional study. We clarified these issues on **pg 5, ln 40 to pg 6, ln 1-4**.
Exclusion criteria: why was the endocrinology subspecialty clinic excluded? what method was used to recruit hospital admin, nurses, residents?	In this study we focused on primary care diabetes care, so we excluded patients seeking care of specialty clinics. We assumed participants referred to endocrine clinic would have either more difficult to control diabetes, more numerous comorbid illnesses, or more complex conditions that would necessitate more specialized care. We included this explanation on **pg 5, ln 21-23**.
Analysis: Likert scales used should be provided as supplementary information	We have included the Likert scales in the submission as **S1 Appendix**.
Figure 2: what is the y -axis of the graph? what is being defined as a challenge?	The y-axis pertains to the proportion of participants reporting challenges on the specific domain of access and component of care. The challenge pertains to the categorization of the Likert scales into problematic as described in **pg 8, ln 18-19**. We clarified this on **Figure 2** by labelling the y-axis and on the Figure caption on **pg 13**.
Table 5: did patients not report covidization of the healthcare system?	The patients also described their experiences of covidization of healthcare without mentioning the actual term, so we did not initially include it on Table 5. However, upon further discussion, we agree to include on the table on **pg 14**.
Discussion: what constitutes a catastrophic health expenditure? while providing great findings, not getting the author's opinion as to why these changes lead to	We operationally defined catastrophic health expenditure as foregoing necessities and/or borrowing

covidization and greater health burdens; what are the root causes that lead to secondary effects exacerbated by the pandemic?	money to afford any component of diabetes care. We clarified this on **pg 8, ln 23-24**.<br><br>We chose to be conservative in presenting our opinion and instead focused on the study results. However, we emphasized how, based on these results, the government's securitized response to the pandemic may have contributed to the challenges in health on **pg 19, ln 36-45**.

In the following table, we describe the changes and justifications for the changes in the reference list. None of the cited papers have been retracted by their respective journals.

Action	Reference	Justification
Removal	World Health Organization. Third round of the global pulse survey on continuity of essential health services during the COVID-19 pandemic [Internet]. Geneva: World Health Organization; 2022 Feb [cited 2023 Feb 27]. Available from: https://www.who.int/publications-detail-redirect/WHO-2019-nCoV-EHS_continuity-survey-2022.1	Part of the introduction that was removed to improve conciseness
Removal	World Health Organization, International Bank for Reconstruction and Development / The World Bank. Tracking Universal Health Coverage: 2021 Global monitoring report [Internet]. Geneva: World Health Organization; 2021 [cited 2022 Dec 10]. Available from: https://www.who.int/publications-detail-redirect/9789240040618	Part of the introduction that was removed to improve conciseness
Addition	Hapal K. The Philippines' COVID-19 Response: Securitising the Pandemic and Disciplining the Pasaway. Journal of Current Southeast Asian Affairs. 2021 Aug 1;40(2):224–44.	Contextualizes the government response to the pandemic
Addition	Paras AY. An Evolutionary Institutionalist Explanation of the Philippines' Militaristic COVID-19 Approach. Soc. 2022 Aug 1;59(4):359–65.	Contextualizes the government response to the pandemic
Addition	Ulep VG, Paterno A, Uy J, Van VS, Casas L, Tan J. The Impact of the COVID-19 Pandemic on Social Health Insurance Claims for High-Burden Diseases in the Philippines [Internet]. Washington DC: Center for Global Development; 2021 May [cited 2023 Jul 20]. Available from: https://www.cgdev.org/publication/impact-covid-19-pandemic-social-health-insurance-claims-high-burden-diseases-philippines	Study on the impact of the pandemic on healthcare utilization for high-burden diseases in the country
Addition	Uy J, Van VTS, Ulep VG, Bayani DB, Walker D. The Impact of COVID-19 on Hospital Admissions for Twelve High-Burden Diseases and Five Common Procedures in the Philippines: A National Health Insurance Database Study 2019-2020. The Lancet Regional Health – Western Pacific [Internet]. 2022 Jan 1 [cited 2023 Jul 20];18. Available from: https://www.thelancet.com/journals/lanwpc/article/PIIS2666-6065(21)00219-4/fulltext	Study on the impact of the pandemic on healthcare utilization for high-burden diseases in the country
Addition	Dworkin SL. Sample Size Policy for Qualitative Studies Using In-Depth Interviews. Arch Sex Behav. 2012 Dec 1;41(6):1319–20.	Previous literature that suggests the use of 30 as ideal sample size in qualitative studies
Addition	Vasileiou K, Barnett J, Thorpe S, Young T. Characterising and justifying sample size sufficiency in interview-based studies: systematic analysis of qualitative health research over a 15-year period. BMC Medical Research Methodology. 2018 Nov 21;18(1):148.	Previous literature that suggests the use of 30 as ideal sample size in qualitative studies
Addition	Guest G, Namey E, Chen M. A simple method to assess and report thematic saturation in qualitative research. PLOS ONE. 2020 May 5;15(5):e0232076.	Expounds on the use of thematic saturation which we used in the qualitative component

We also report the changes we implemented in the figures and corresponding justifications.

<table>
<tr><th>Fig</th><th colspan="2">Description of Change</th><th>Justification</th></tr>
<tr><td rowspan="2">1</td><td>Original Submission
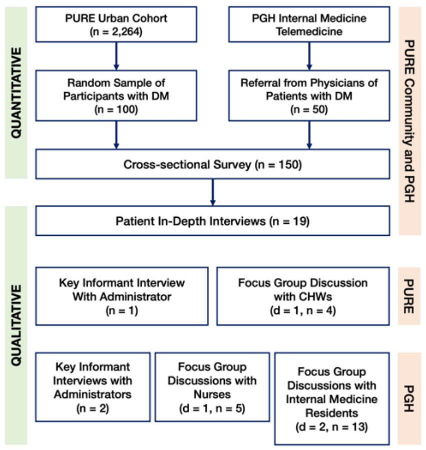</td><td>Revised Submission
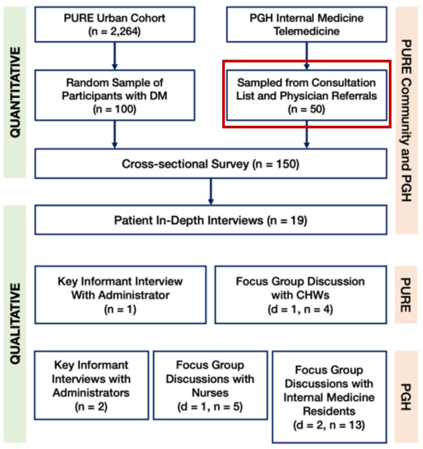</td><td rowspan="2">The current figure more accurately reflects the recruitment process at PGH OPD (n.b., this was already described in the original submission, see pg 5, ln 36-40 of original manuscript and pg 5, ln 40 to pg 6, ln 4 of the revised manuscript).</td></tr>
<tr><td colspan="2">More complete description of recruitment strategy at PGH OPD</td></tr>
<tr><td rowspan="2">2</td><td>Original Submission
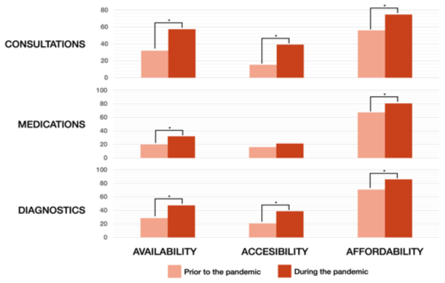</td><td>Revised Submission
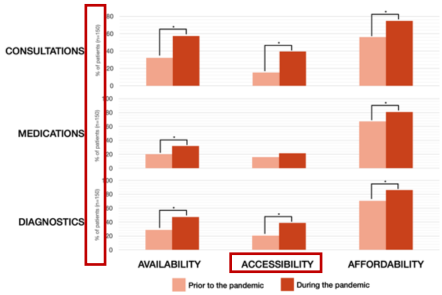</td><td rowspan="2">The current figure adds clarity on what the numbers on the y-axis mean.</td></tr>
<tr><td colspan="2">Addition of y-axis labels and correction of spelling error</td></tr>
</table>

Finally, we updated the pagination of the STROBE and SRQR checklists to accurately reflect the latest manuscript.

We hope that these responses adequately address the comments and add clarity and quality to our submission. Please address all correspondence concerning this manuscript to **lijing.yan@duke.edu**.

Thank you once again for your kind consideration.

Sincerely,

Greco Mark B. Malijan, MD, MSc

On behalf of Prof. Lijing L. Yan (corresponding author)
