# Supplementary material for: Assessing COVID-19 pandemic’s impact on essential diabetes care in Manila, the Philippines: A mixed methods study
Source: PLOS Glob Public Health. 2024 Jan 23;4(1):e0002333. doi: 10.1371/journal.pgph.0002333 (PMC10805280; doi:10.1371/journal.pgph.0002333)
Supplement: Attachment — Submitted filename: Response to Reviewers.pdf [file pgph.0002333.s009.pdf]

30 November 2023

Prof. Catherine Kyobutungi
Prof. Madhukar Pai
Editors-in-Chief
PLOS Global Public Health

Through: **Dr Rajat Das Gupta**
Academic Editor
PLOS Global Public Health

We thank the editors and reviewers for taking great interest in our work and for providing important and actionable feedback that we hope will help improve our manuscript and make our article meet the current journal standards.

In the following table, we respond to the comments and highlight where we implemented the changes in the revised submission.

Comments	Authors' Responses
**Journal Requirements**	
Please review your reference list to ensure that it is complete and correct. If you have cited papers that have been retracted, please include the rationale for doing so in the manuscript text, or remove these references and replace them with relevant current references. Any changes to the reference list should be mentioned in the rebuttal letter that accompanies your revised manuscript. If you need to cite a retracted article, indicate the article's retracted status in the References list and also include a citation and full reference for the retraction notice.	We have not altered our reference list.
Please provide separate figure files in .tif or .eps format only and remove any figures embedded in your manuscript file. Please also ensure all files are under our size limit of 10MB.	We have uploaded separate figure files as .tif in accordance with the image guidelines and have removed any figures embedded in the main manuscript. We used the suggested PACE tool to generate the figure files.
We have noticed that you have uploaded Supporting Information files, but you have not included a list of legends. Please add a full list of legends for your Supporting Information files after the references list.	We have included the Supporting Information caption in the manuscript.
**Reviewer #1**	
The authors have addressed the reviewer's comments well, and comprehensively. The paper provides a mixed methods approach to understanding healthcare systems access among those with diabetes during the COVID 19 pandemic. The paper is well-presented, well-written, and provides important findings that likely have global relevance. Additionally, the authors provide to the scientific community an excellent example of how to use a mixed methods approach (and specifically an explanatory sequential model) to garner and obtain a nuanced understanding of the health issues and healthcare access among this sample of participants. It is recommended that the paper be accepted for publication.	We thank you for your review and recommendation.
**Reviewer #3**	
The manuscript demonstrates commendable comprehensiveness and structural organization. However, some critical queries remain.	We thank you for your helpful review and recommendations. We have addressed your concerns accordingly.

Clarifications are needed regarding the rationale behind exclusion criteria such as "Unable to give informed consent" and "Seeking care at a specialty clinic for diabetes care."	We believe that an individual's inability to provide informed consent to be a necessary criterion for exclusion; hence, we specified it in our study protocol and in the manuscript. Moreover, we have provided an explanation in the manuscript why we excluded those seeking care at specialty clinic (i.e., we assumed that patients referred to the specialty clinic were more likely to have more complex medical conditions necessitating specialized care beyond the level of primary care). See **page 5, lines 22-27**.
The recruitment section should provide numerical insights into the distribution of participants across different recruitment methods (PURE study, PGH OPD telemedicine, physician referrals) and their representativeness vis-à-vis the entire population.	We would like to seek clarification on this question. We have previously stated in the manuscript and showed in figure 1 that we used stratified random sampling to generate 100 patients with diabetes from the PURE urban cohort which at the time had 2,264 participants. See **Fig 1**.
A rationale for the relevance of "catastrophic health expenditure" to the study's objectives should be expounded.	As stated in the Introduction section, when we designed and implemented the study, our aim was to describe how diabetes care, as a tracer condition for NCDs in general, was affected by the pandemic. We adapted the Levesque framework to study the phenomenon, and catastrophic health expenditure is strongly related to the affordability of diabetes care. We have provided additional explanation on of its relevance on **page 6, lines 39-42**.
Address the potential sampling bias introduced by including patients from PGH OPD and the community cohort, emphasizing their limited representativeness of the broader diabetes patient population in the Philippines.	We have previously specified potential biases related to our sampling strategy as second and third limitations in the discussion section of the manuscript. See **page 18, line 42 to page 19 line 11**.
Thoroughly discuss the limitations associated with recall and social desirability biases, elucidating their potential impact on result validity and the mitigation strategies employed.	We included more details regarding these biases as part of the fourth limitation in the discussion section of the manuscript. See **page 19, lines 13-25.**
In the conclusion, consider incorporating a brief discussion of potential policy implications and recommendations to guide future actions in tackling the highlighted challenges.	We have considered this recommendation greatly, and we agree with the reviewer about the need to highlight how our findings can guide future actions. In the end we have decided to be conservative in what we include in the Conclusion section and chose to highlight the key findings of our study. However, we do briefly touch on the need to prioritize health financing strategies, especially for NCDs that require continuous routine outpatient care, in preparing for the next pandemic both in the Discussion section and in the Conclusion. See **page 18, lines 22-31 and page 19, line 43 to page 20 line 2**.
**Reviewer #4**	
Thank you for the opportunity to review the manuscript. It is nicely done.	We thank you for your review and recommendation.
**Reviewer #5**	
The study by Malijan et al investigated pandemic's Impact on essential diabetes care in Manila. They found that in their country pandemic negatively impacted access to essential diabetes care.<br>*Comments and criticisms. A number of similar studies has been published on the subject in 2021-2022 and the focus on the problem has significantly diminished. I do not think that the present paper will be found of interest by most readers. Maybe a local journal could me the right target. Furhermore the authors themselves underlined the*	We thank you for your review and assessment. It is unfortunate that the reviewer does not think that the present paper will be of interest to most readers of PLOS Global Public Health, but also appreciate that other reviewers have recommended that the paper be accepted for publication in the journal.<br>We have developed the manuscript with close attention to the study's limitations and have taken into consideration other reviewer comments that required

weaknesses of the study, of which the most significant is the small sample size: Manila is a city with millions of inhabitants and reporting the interview of single patients, as reported, is of little meaning.<br>*The simple conclusions are expected and most of the manuscript (too long!) is represented by the method section. Also the introduction is unecessarily long.*	us to be more detailed in the introduction and methods sections.<br>We agree that the sample size for the quantitative component is small and have already discussed this at length as part of limitations. However, we do not agree that "reporting the interview of single patients, as reported, is of little meaning." We argue that the goal of the qualitative component of this mixed method study is not necessarily generalizability but transferability of the findings within the wider population and to other similar populations. The reporting of these qualitative findings provides nuance to the experiences by Filipinos living with diabetes in Manila during the pandemic, and we deem it necessary to share these findings with a wider readership. Part of the reasons why the method section is lengthy is to reflect the ways in which we as researchers show credibility in this work (e.g., reflexivity and positionality), so it is unfortunate the reviewer did not appreciate our approach.
**Reviewer #7**	
It is a well written article but lacks the information on how the country's policy on containing the COVID virus, or minimalizing the spread was enforced or how the system was functioning during lockdown and entire pandemic session.	We thank you for your helpful review. We have previously focused the third paragraph of our Introduction section to give context on the government's response to the pandemic. We also mentioned different studies supporting the relationship between lockdown policy implementation and healthcare access limitations. See **page 3 lines 6-19**.
Self diabetic care at home by glucometer and how many of the small sample size you have were using it?	This is an important suggestion, and we thank the reviewer for this comment. Unfortunately, our quantitative instrument did not explicitly capture the number/proportion of those who used glucometer at home; hence, we cannot provide it in our discussion.
What would have been better is if the solution to that was also discussed in KII or at least mention the strategies/methods of preparedness before another lockdown hits in Discussion part. It is also worth mentioning how the country is otherwise managing NCDs. Does it have a free distribution of medicines for already diagnosed diabetic or hypertensive patients who fall under the category of earning <5000/monthly PHP. If the country is making a policy to reduce NCDs, how is it planning for it?	This is another important suggestion from the reviewer, but unfortunately, as shown in our interview guides (**see S2 Appendix)**, our KII with administrators focused on key institutional changes to diabetes care provision and the challenges they faced, which limits our ability to provide comprehensive answer to the question of the reviewer. In the study setting component of the methods section, we added more details regarding distribution of medications in local health centers. Briefly, selected medications including those for hypertension and diabetes are provided free at point of care regardless of household income, but the availability of these medications are dependent on the financial capabilities of the local government unit overseeing the health centers. See **page 4, lines 34-37**.

We hope that these responses adequately address the comments and add clarity and quality to our submission. Please address all correspondence concerning this manuscript to **lijing.yan@duke.edu**.

Thank you once again for your kind consideration.

Sincerely,

Greco Mark B. Malijan, MD, MSc

On behalf of Prof. Lijing L. Yan (corresponding author)
